# An improved rhythmicity analysis method using Gaussian Processes detects cell-density dependent circadian oscillations in stem cells

**DOI:** 10.1093/bioinformatics/btad602

**Published:** 2023-09-28

**Authors:** Shabnam Sahay, Shishir Adhikari, Sahand Hormoz, Shaon Chakrabarti

**Affiliations:** Department of Computer Science, Indian Institute of Technology Bombay, Mumbai, Maharashtra 400076, India; Simons Centre for the Study of Living Machines, National Centre for Biological Sciences, Bangalore, Karnataka 560065, India; Department of Systems Biology, Harvard Medical School, Boston, MA 02215, United States; Department of Data Science, Dana-Farber Cancer Institute, Boston, MA 02215, United States; Department of Systems Biology, Harvard Medical School, Boston, MA 02215, United States; Department of Data Science, Dana-Farber Cancer Institute, Boston, MA 02215, United States; Broad Institute of MIT and Harvard, Cambridge, MA 02142, United States; Simons Centre for the Study of Living Machines, National Centre for Biological Sciences, Bangalore, Karnataka 560065, India

## Abstract

**Motivation:**

Detecting oscillations in time series remains a challenging problem even after decades of research. In chronobiology, rhythms (for instance in gene expression, eclosion, egg-laying, and feeding) tend to be low amplitude, display large variations amongst replicates, and often exhibit varying peak-to-peak distances (non-stationarity). Most currently available rhythm detection methods are not specifically designed to handle such datasets, and are also limited by their use of *P*-values in detecting oscillations.

**Results:**

We introduce a new method, ODeGP (**O**scillation **De**tection using **G**aussian **P**rocesses), which combines Gaussian Process regression and Bayesian inference to incorporate measurement errors, non-uniformly sampled data, and a recently developed non-stationary kernel to improve detection of oscillations. By using Bayes factors, ODeGP models both the null (non-rhythmic) and the alternative (rhythmic) hypotheses, thus providing an advantage over *P*-values. Using synthetic datasets, we first demonstrate that ODeGP almost always outperforms eight commonly used methods in detecting stationary as well as non-stationary symmetric oscillations. Next, by analyzing existing qPCR datasets, we demonstrate that our method is more sensitive compared to the existing methods at detecting weak and noisy oscillations. Finally, we generate new qPCR data on mouse embryonic stem cells. Surprisingly, we discover using ODeGP that increasing cell-density results in rapid generation of oscillations in the *Bmal1* gene, thus highlighting our method’s ability to discover unexpected and new patterns. In its current implementation, ODeGP is meant only for analyzing single or a few time-trajectories, not genome-wide datasets.

**Availability and implementation:**

ODeGP is available at https://github.com/Shaonlab/ODeGP.

## 1 Introduction

From the rapid ultradian oscillations of p53, NF-κB, Hes7, and the embryonic segmentation clock, to the slower seasonal flowering patterns in plants—oscillations in biological systems are ubiquitous across many length and time scales (Kruse and Jülicher 2005, Cao *et al.* 2016). These patterns, where repeatedly occurring peaks can be observed in the time series of interest, are often noisy, exhibit low amplitudes (peak-to-trough distance), and are non-stationary (peak-to-peak distance varies with time). Classic examples of such noisy oscillations can be observed in circadian clock gene expression (So *et al.* 2009, Phillips *et al.* 2021), feeding, eclosion, and egg-laying rhythms (Xu *et al.* 2008, Menon *et al.* 2014, Nikhil *et al.* 2016). This makes it hard to distinguish rhythmic patterns from noise, necessitating the development of quantitative approaches to accurately infer the existence of oscillations, and subsequently extract parameters, such as time period and amplitude (Refinetti *et al.* 2007). Developing principled approaches to detecting oscillations is also important in differential rhythmicity analyses, where genes can lose or gain rhythmicity after perturbations (Thaben and Westermark 2016, Singer and Hughey 2019, Pelikan *et al.* 2021). Finally, biological oscillators are often coupled, and investigating the nature and consequences of coupling often depends on the ability to carefully measure and detect the individual oscillations in the first place (Hannay *et al.* 2018, Chakrabarti and Michor 2020, Heltberg *et al.* 2021).

Over the last few decades, numerous methods have been developed to address the oscillation-detection problem. Existing non-parametric methods used for oscillation detection include JTK_Cycle (Hughes *et al.* 2010) and RAIN (Thaben and Westermark 2014). eJTK (Hutchison *et al.* 2015) is a more recently developed algorithm that improves on JTK_Cycle by including non-sinusoidal reference waveforms. MetaCycle (Wu *et al.* 2016), an R package developed to identify oscillations, integrates the results of JTK_Cycle, the Lomb–Scargle periodogram (Scargle 1982) (a parametric method) and ARSER (Yang and Su 2010) (a parametric method using autoregressive models, which cannot work on unevenly sampled data). Another existing R package for oscillation detection is DiscoRhythm (Carlucci *et al.* 2019), which builds on MetaCycle by additionally using the Cosinor (Cornelissen 2014) method (also parametric). Among the non-parametric methods, RAIN requires that the period to test for (or a range of periods) be specified beforehand when trying to identify oscillations. Similarly, JTK_Cycle and MetaCycle also require information about the expected oscillation time period to be provided as inputs to the algorithm. More recently, neural network-based approaches have been used for classifying oscillatory versus non-oscillatory datasets (Agostinelli *et al.* 2016), but these do not allow learning of the full waveform, besides requiring a lot of training data to perform well. Finally, wavelet-based techniques (Leise and Harrington 2011, Mönke *et al.* 2020) can extract time-dependent phase and periods from temporal datasets, but are not designed to specifically detect oscillations or handle replicate data. More details and comparisons of a variety of oscillation-detection algorithms can be found elsewhere—Refinetti *et al.* (2007) provides a comprehensive review of earlier techniques while comparisons of more recent methods can be found in Laloum and Robinson-Rechavi (2020), Mei *et al.* (2020), Wu *et al.* (2014), Deckard *et al.* (2013), and Hughes *et al.* (2017).

While these methods have improved over time and have become increasingly powerful, a number of challenges in oscillation detection are yet to be overcome (Hughes *et al.* 2017)—(i) data are often available only at non-evenly spaced time points, which can make oscillation detection difficult, particularly with Fourier Transform based methods, (ii) large error bars at each time point from replicates are often not easy to incorporate into existing methods, (iii) biological oscillations tend to be non-stationary (peak-to-peak distance varies over time), which parametric models cannot handle well due to difficulty in defining functional forms for such data, and finally (iv) most current methods rely on calculating a *P*-value to classify a dataset as rhythmic versus non-rhythmic [for an exception, see Obodo *et al.* (2023)], but a major issue with *P*-value-based approaches is that they model only the null, but not the alternative hypothesis (Hughes *et al.* 2017, Hutchison and Dinner 2017, Benjamin and Berger 2019, Halsey 2019). Existing methods often overcome one or few of these challenges, but no single method exists that addresses all these problems in a comprehensive manner.

To solve the above four challenges in a unified framework, here, we develop ODeGP (**O**scillation **De**tection using **G**aussian **P**rocesses), a new approach to the oscillation-detection problem combining Gaussian Process (GP) regression (Rasmussen and Williams 2005) with Bayesian model selection. Conceptually, the non-parametric nature of GPs allows ODeGP to flexibly model both stationary and non-stationary datasets, while the Bayesian model selection approach using Bayes factors allows us to model both the null as well as alternate hypotheses, unlike *P*-value-based methods. In particular, we use a recently developed non-stationary kernel that allows us to model non-stationary datasets (Heinonen *et al.* 2015, Remes *et al.* 2017), improving the accuracy of oscillation detection over many of the popular existing methods, such as eJTK and MetaCycle. Additionally, ODeGP provides a simple Bonferroni-type multiple-hypothesis correction (Westfall *et al.* 1997), though this approach currently limits its use to settings where only one or a few trajectories are to be analyzed, not genome-wide datasets. Given the flexibility and power of GPs, there has been surprisingly limited use in analyzing biological datasets. Our work adds to the few prior examples, for instance in identifying differentially expressed genes (Heinonen *et al.* 2015, Topa and Honkela 2018), detecting oscillations (Durrande *et al.* 2016, Phillips *et al.* 2017), and the discovery of spatial patterns in gene expression (Svensson *et al.* 2018).

We extensively compare the performance of ODeGP with eight other existing methods on both simulated and experimental datasets and demonstrate that it is consistently better and more sensitive at identifying oscillations. We also find that the Bayes factor usually has a large separation between oscillatory and non-oscillatory experimental datasets, suggesting that it is a good metric for the classification problem. Finally, to test the usefulness of ODeGP in learning patterns in new datasets, we generate time-series circadian clock gene expression profiles using mouse embryonic stem cells (mESCs). Intriguingly, we find that oscillations of *Bmal1* can be induced within a few days in mESCs by increasing cell density and that these oscillations get suppressed with the addition of MEK/ERK and GSK3b inhibitors. This interesting result adds to previous observations that while pluripotent mESCs exhibit no clock gene oscillations, retinoic acid (RA)-mediated differentiation can induce oscillations after about 2 weeks (Yagita *et al.* 2010, Umemura and Yagita 2020). Our results indicate that increasing cell density might mimic the effects of directed differentiation, but with faster kinetics of emergence of circadian gene oscillations.

## 2 Materials and methods 

### 2.1 ODeGP—GP regression for oscillation detection

A brief mathematical introduction to the theory of GPs is provided in Supplementary Section S3 and full details of ODeGP are provided in Supplementary Section S4. Here, we provide a brief overview of the algorithm. ODeGP uses two different kernel functions, $K_{D}$ and $K_{\text{NS}}$, to generate two GP priors. The diagonal kernel $K_{D}(x,x^{\prime })=\epsilon^{2}\cdot \delta_{xx^{\prime }}$ is used to represent non-oscillatory functions, where δ is the Kronecker delta. This kernel encodes the prior belief that there is no correlation between the values of the data at different time points, thus all the non-diagonal entries of the covariance matrix are zero. To represent oscillatory functions, the non-stationary kernel $K_{\text{NS}}(x,x^{\prime })=w(x)w(x^{\prime })k_{\text{gibbs}}(x,x^{\prime })\;\;\text{cos}(2\pi (x\mu (x)-x^{\prime }\mu (x^{\prime }))+\epsilon^{2}\cdot \delta_{xx^{\prime }}$ is used, where $k_{\text{gibbs}}$ is the Gibbs kernel. After the data are obtained, Bayes theorem allows construction of the posteriors where the optimal hyperparameters for the non-stationary and diagonal kernels are learnt through maximization of the marginal log-likelihoods (MLLs). The ratio of the MLLs corresponding to $K_{\text{NS}}$ and $K_{D}$ gives the Bayes factor, which provides the degree of evidence for presence of oscillations. Finally, ODeGP also provides a Bonferroni-type multiple-hypothesis correction term via a prior odds that is multiplied with the Bayes factor.

### 2.2 Mathematical modeling to investigate amplitude and coupling effects on the Bayes factor

We used the Poincare model of coupled phase-amplitude oscillators (Schmal *et al.* 2018) to investigate how the Bayes factor from ODeGP is affected by increasing the amplitude of single cell oscillators as well as coupling between them. The basic equations in Cartesian coordinates $x_{i},y_{i}$ (with $r_{i}=\sqrt{x_{i}^{2}+y_{i}^{2}}$) are provided here; for more details on the integration scheme and parameter values see Supplementary Section S9.


(1) $$\begin{array}{c} \frac{dx_{i}}{dt}=\gamma_{i}x_{i}(A_{i}-r_{i})-\frac{2\pi }{\tau_{i}}y_{i}+M,\\ \frac{dy_{i}}{dt}=\gamma_{i}y_{i}(A_{i}-r_{i}), \end{array}$$

where the mean field $M=\frac{K}{N}\sum_{i=1}^{N}x_{i}(t)$, and *N* is the total number of single cell oscillators, which are coupled via the mean field *M*. The coupling strength is parameterized by *K*, the free-running time period, amplitude and amplitude relaxation rate of the *i*th oscillator are $\tau_{i}$, $A_{i}$, and $\gamma_{i}$, respectively (Schmal *et al.* 2018).

### 2.3 Cell lines and culture conditions

A passage 12 (p12) mESC line (E14TG2a.4) was expanded under conditions expected to maintain pluripotency, and all cells used for the experimental data reported here were within 10 additional passages. The p12 cells were thawed and expanded on 0.1% gelatin-coated, cell-culture treated 10 cm plastic dishes. GTES ES cell media (GMEM, 15% FBS, Glutamax, sodium pyruvate, non-essential amino acids, BME, and LIF) was used to propagate the cells in pluripotent conditions. Cells were passaged at ∼70% confluency to avoid crowding-induced differentiation. For all experiments that required thawing out new vials, the cells were always initially maintained in cell-culture treated plastic dishes coated with 0.1% gelatin, and then transferred to various other conditions, such as glass dishes with fibronectin or laminin coating.

### 2.4 qPCR experiments—data collection protocol and error analysis

For the qPCR experiments, cells were collected in Trizol, total RNA was extracted and converted to cDNA, and finally, qPCR was performed using the SYBR Green dye. Each time point (beginning from time zero, corresponding to immediately after Dex synchronization) corresponds to cells obtained from an independent well of a 24-well plate. To avoid any artifacts in cell synchronization due to differences in cell density at different times of cell collection, we devised a protocol to ensure an approximately equal number of cells collected for every time point: instead of synchronizing all the samples at one-time point and collecting cells at different time points, we synchronized cells at different times and Trizol collected the cells at a single time. Details of the protocol, seeding densities, and various substrate conditions for the samples are provided in Supplementary Section S1. The error analysis is explained in Supplementary Section S2.

## 3 Results

### 3.1 ODeGP—an oscillation-detection algorithm based on GPs

**Figure 1.**
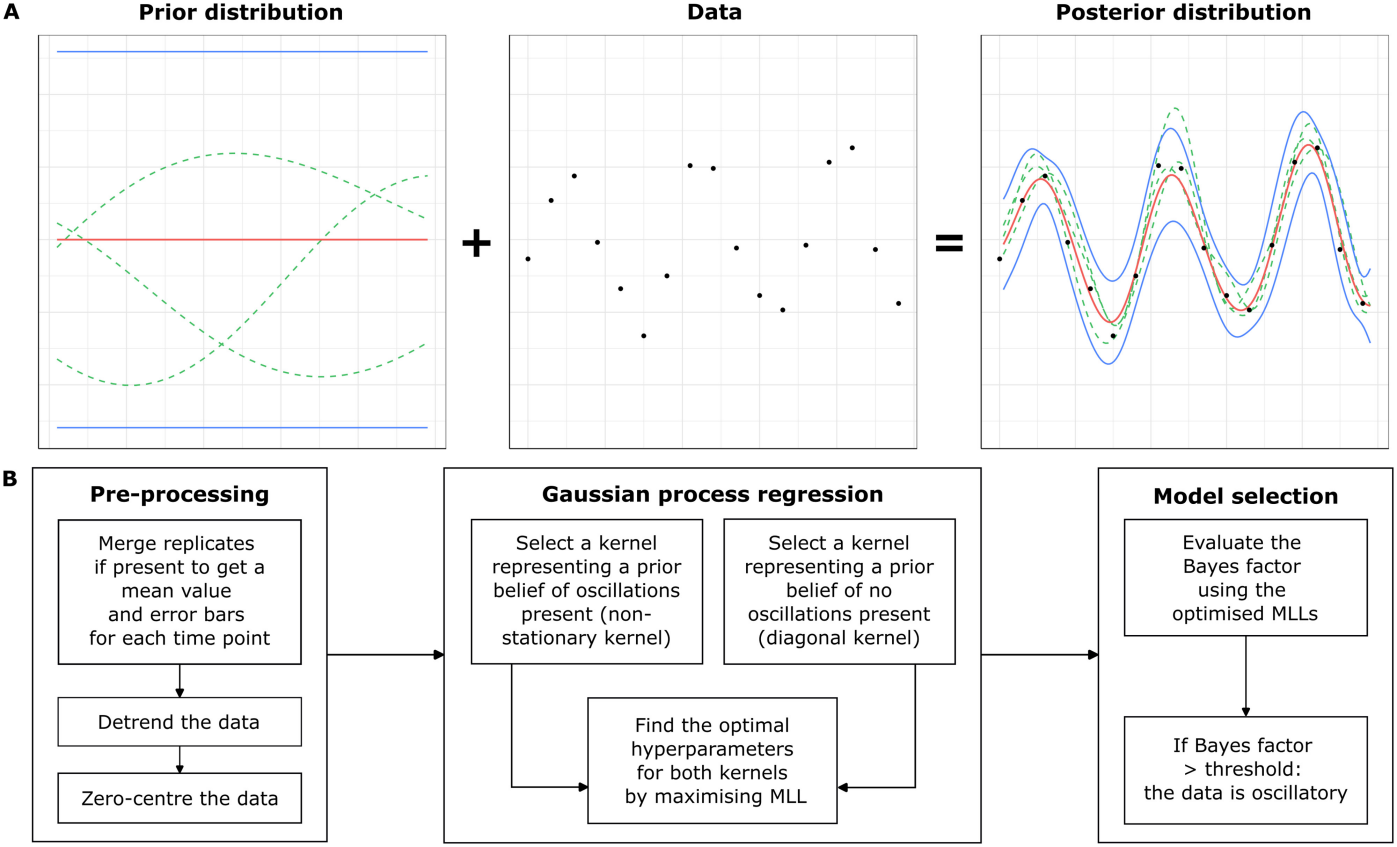
An overview of ODeGP’s workflow. (A) An intuitive schematic of GP regression, where a GP is an indexed collection of random variables with a multivariate normal joint probability density. Left: a GP prior defining a distribution over functions (instantiations of the functions are shown in green lines; mean and confidence intervals are in red and blue, respectively). Middle: observed data. Combining the observed data and the GP prior allows the construction of the data likelihood. Right: posterior distribution generated after Bayesian inference, which models the given data closely. Green lines are instantiations of the posterior, red and blue lines are the posterior mean and standard deviation, respectively. (B) The workflow of ODeGP. The pre-processing stage involves formatting and normalizing the data. Next, two separate regressions are performed with the diagonal and non-stationary kernels, respectively. The optimized MLL of the data is found for each case. These MLL values are then used to compute the Bayes factor for model selection. This Bayes factor is the final output metric used to determine whether the data are oscillatory or not.

We developed a new method for detecting oscillations, ODeGP (**O**scillation **De**tection using **G**aussian **P**rocesses), combining GP regression and Bayesian inference. A brief intuition of how GP regression works is provided in Fig. 1A and an outline of the ODeGP pipeline is displayed in Fig. 1B. In brief, GP regression is a non-parametric approach to learning non-linear trends in data, where instead of specifying a function and learning its optimal parameters (parametric regression), the functional form itself is learnt by specifying a prior over functions (Rasmussen and Williams 2005). Placing a prior over functions is achieved by the use of a multivariate Gaussian (Fig. 1A, left), whose covariance matrix Σ is determined by a kernel that prioritizes certain classes of functions based on prior expectations of smoothness and characteristic length-scales associated with the problem of interest (see Section 2 and Supplementary Sections S3 and S4 for details). After data are obtained, the instantiations of the prior that best describe the data are obtained via the posterior (Fig. 1A, right), which can be described via the posterior mean and variance. Model complexity is represented by the determinant of Σ, which can penalize an increase in the number of kernel hyperparameters and thus prevent overfitting of the data. A more detailed analysis of this is presented in Supplementary Section S6, Supplementary Fig. S1, and Supplementary Table S7. Finally, the possibility of performing corrections for multiple-hypothesis testing is also provided (see Section 2 and Supplementary Section S4).

We generated a wide variety of simulated datasets where the ground truth (oscillatory or non-oscillatory) is known. These datasets are summarized in Table 1, and comparisons of various methods on these datasets are discussed in the next sections (see also Supplementary Section S5). To mimic experimental qPCR data, all simulated waves were generated as sets of three replicates, with a total duration of 48 h and an interval of 3 h between consecutive observations. Two relative levels of noise (low and high, corresponding to σ=0.1 and σ=1, respectively), along with two different fractions of missing data (none and half, with the time points for missing data selected randomly), were used to create further variation among the datasets. Finally, three different ways of generating non-stationary data were tested (details in Supplementary Section S5).

**Table 1.** Categories of simulated data used for comparing the performance of existing oscillation-detection methods with ODeGP.

All datasets: 3 replicates, 48 h duration, 3 h spacing		
Noise level: low/high, fraction of missing data: 0/0.5		
Simulated non-oscillatory datasets	Gaussian noise (random sample from N(0,σ) at each time point)	
Simulated oscillatory datasets	Stationary symmetric data (combinations of sine waves)	Stationary asymmetric data (sawtooth waves)
	Non-stationary symmetric data (continuously decreasing or randomly varying time period)	Non-stationary asymmetric data (randomly varying time period)

### 3.2 Detecting oscillations in simulated stationary datasets

We first tested ODeGP on simulated stationary data. Datasets consisting of both non-oscillatory and stationary oscillatory waves were generated, and the ability of each method to distinguish the two was evaluated through the construction of receiver operating characteristic (ROC) curves.

**Figure 2.**
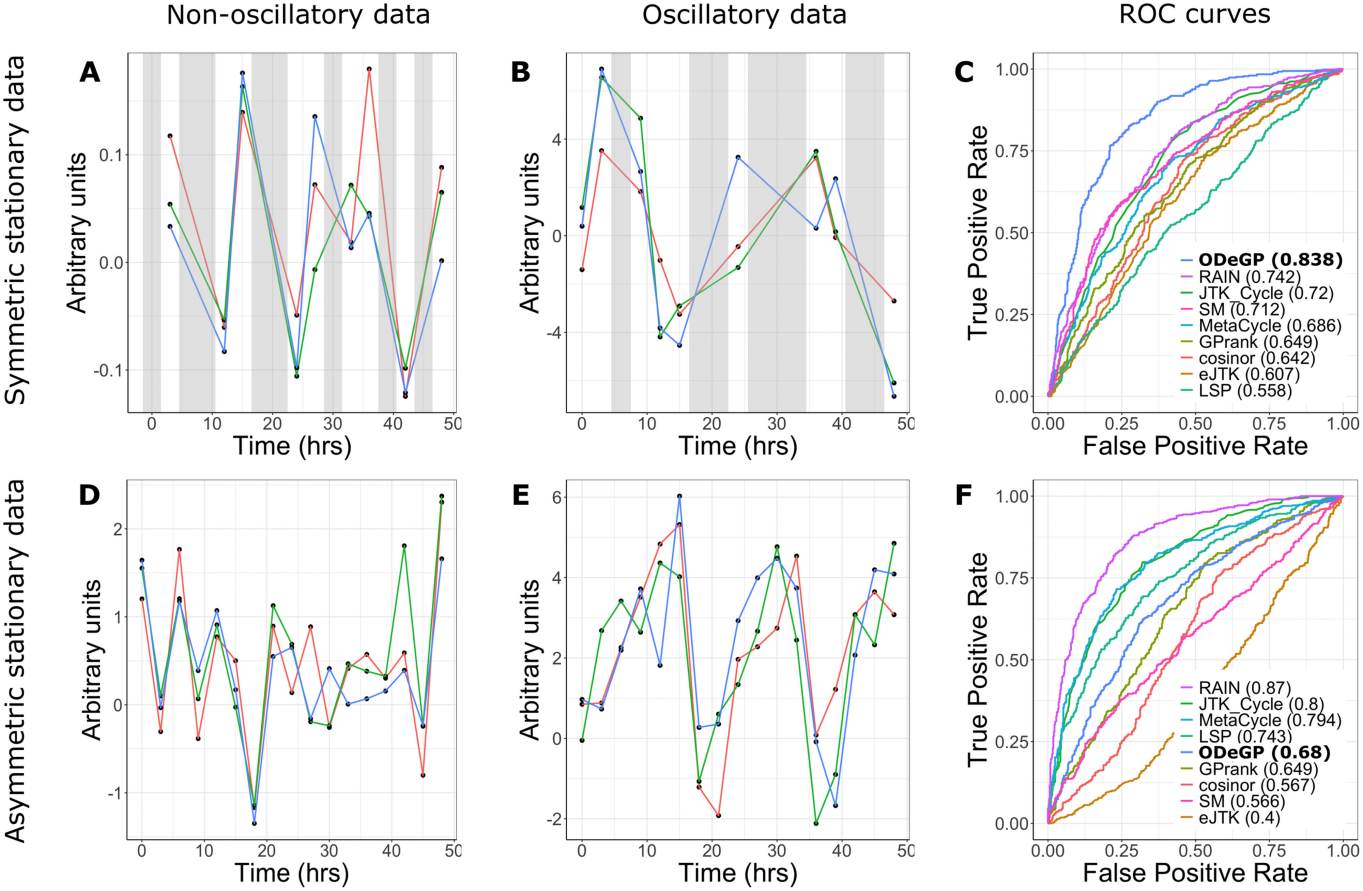
Detecting oscillations in simulated stationary datasets. All waves shown are sets of three replicates. (A) Simulated non-oscillatory data with σ=0.1 and half the points missing. (B) Simulated symmetric stationary data with $A_{1}=A_{2}=3,\tau_{1}=18,\tau_{2}=26$, σ=0.1 and half the points missing. (C) ROC curves for all methods considered on the dataset consisting of waves generated like (A) and (B). Numbers in brackets denote AUC values. (D) Simulated non-oscillatory data with σ=1 and no points missing. (E) Simulated asymmetric stationary data with A=5,τ=18 and σ=1. (F) ROC curves for all methods considered on the dataset consisting of waves generated like (D) and (E). Grey shaded areas represent regions with missing data. LSP, Lomb–Scargle periodogram; SM, spectral mixture kernel.

Non-oscillatory waves were simulated by randomly sampling from a standard normal distribution with standard deviation σ at each time point in consideration: f(t)=N(0,σ), as shown in Fig. 2A. The three replicates are shown with red, blue, and green lines. Symmetric stationary waves, as in Fig. 2B, were generated by the addition of two sine waves: $f(t)=A_{1}\;\text{sin}(2\pi t/\tau_{1})+A_{2}\;\text{sin}(2\pi t/\tau_{2})+N(0,\sigma )$.

The performance of various methods in terms of correctly identifying the presence of oscillations in symmetric stationary waves was evaluated by generating ROC curves on a collection of 500 oscillatory and 500 non-oscillatory waves. These ROC curves for data corresponding to the waves in Fig. 2A and B are shown in Fig. 2C. ODeGP performs the best on this set of 1000 waves, with an AUC value of 0.838. AUC values for all other datasets are reported in Supplementary Section S5.

Similarly, performance on asymmetric stationary oscillatory waves was evaluated by generating ROC curves on a collection of 500 non-oscillatory waves (as shown in Fig. 2D) and 500 asymmetric stationary oscillatory waves. The latter were generated using the functional form of sawtooth waves: $f(t)=A\cdot \{\frac{t}{\tau }\}+N(0,\sigma )$, where {} represents the fractional part function. Figure 2E shows a wave generated as such. RAIN performs the best on this set of 1000 waves as seen in Fig. 2F with an AUC value of 0.87, while the non-stationary kernel has a relatively poorer AUC value of 0.68.

The relative performance of the methods tested on all stationary datasets generated is summarized in Table 2 (all AUC values are provided in Supplementary Section S5). ODeGP is consistently among the best three performing methods in all symmetric stationary datasets considered. Cosinor and eJTK, which are among the best three methods the second-most times, also fall among the worst three methods a significant number of times. In asymmetric stationary datasets, RAIN is among the best three methods the most consistently, whereas ODeGP here has a relatively average performance (neither being many times among the best three nor many among the worst three). RAIN’s better performance compared with our method on these datasets is expected because it separately groups the rising and falling parts of the waves for comparison, boosting its ability to identify asymmetric waveforms (Thaben and Westermark 2014).

**Table 2.** Comparison of the number of times each method tested appears among the best and worst performing three methods, in all stationary datasets considered.^a^

Stationary datasets				
Method	Symmetric datasets (total: 9)		Asymmetric datasets (total: 8)	
	Times among best three methods	Times among worst three methods	Times among best three methods	Times among worst three methods
Cosinor	5	3	3	4
eJTK	5	4	4	4
GPrank	0	6	0	5
JTK_Cycle	1	0	3	0
LSP	0	6	1	4
MetaCycle	1	0	4	0
ODeGP	9	0	2	1
SM Kernel	2	5	0	6
RAIN	4	3	7	0

a LSP, Lomb–Scargle periodogram; SM, spectral mixture.

### 3.3 Detecting oscillations in simulated non-stationary datasets

Since experimental time-series qPCR data tend to be non-stationary (peak-to-peak distance varies with time), we next tested the ability of each method to distinguish non-stationary oscillatory waves from non-oscillatory waves.

**Figure 3.**
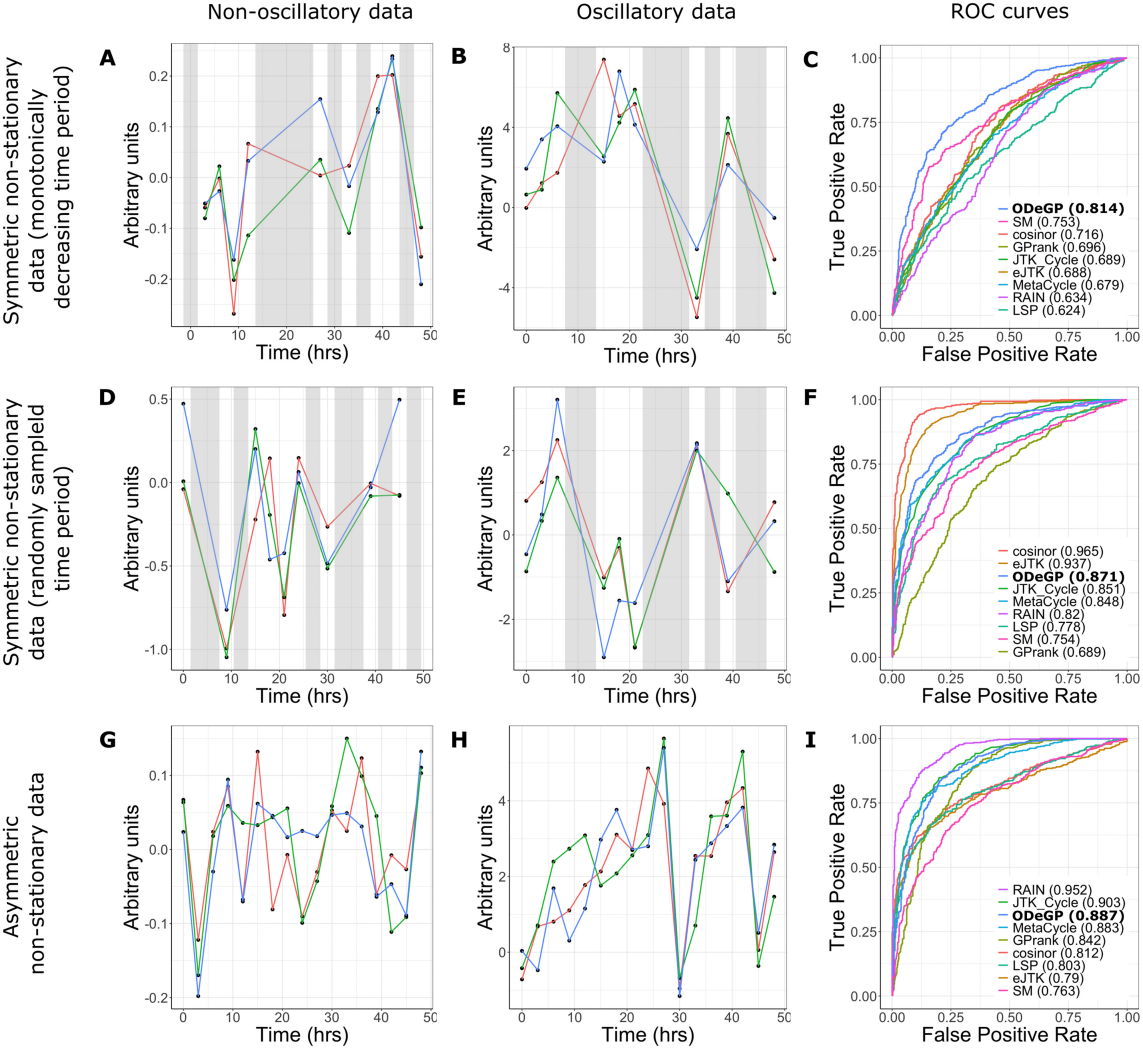
Detecting oscillations in simulated non-stationary datasets. All waves shown are sets of three replicates. (A) Simulated non-oscillatory data with σ=0.1 and half the points missing. (B) Simulated symmetric non-stationary data with a monotonically decreasing time period, with A=5,τ=72,σ=0.1 and half the points missing. (C) ROC curves for all methods tested on the dataset consisting of waves generated like (A) and (B). (D) Simulated non-oscillatory data with σ=0.5 and half the points missing. (E) Simulated symmetric non-stationary data with a randomly varying time period, with A=1.5,μ=24,σ=1.33,σ=0.5, and half the points missing. (F) ROC curves for all methods tested on the dataset consisting of waves generated like (D) and (E). (G) Simulated non-oscillatory data with σ=0.1 and no points missing. (H) Simulated asymmetric non-stationary data with $A=5,\tau_{1}=12,\tau_{2}=30$, and σ=0.1. (I) ROC curves for all methods tested on the dataset consisting of waves generated like (G) and (H). Grey shaded areas represent regions with missing data. Numbers in brackets in (C), (F), and (I) correspond to AUC values. LSP, Lomb–Scargle periodogram; SM, spectral mixture kernel.

Symmetric non-stationary oscillatory waves with a monotonically decreasing time period, as shown in Fig. 3B, were generated using the functional form $f(t)=A\cdot \;\text{sin}(\frac{2\pi t}{1+|t-\tau |})+N(0,\sigma )$, where τ≥48. The ROC curves in Fig. 3C indicate that ODeGP performs the best on a dataset consisting of 500 non-oscillatory waves generated like in Fig. 3A and 500 symmetric non-stationary oscillatory waves generated like in Fig. 3B, with an AUC value of 0.814.

Since there can be many ways of generating non-stationary data, an additional approach to generating time-varying periodicities in a single wave was explored. Symmetric oscillatory waves were generated with a randomly varying time period instead of a monotonically decreasing one, as follows: at the start of each new oscillation, a time period τ is sampled from N(μ,σ). If the total time elapsed up to the start of this new oscillation is ϕ, then $f(t)=A\cdot \;\text{sin}(\frac{2\pi (t-\phi )}{\tau })+N(0,\sigma )$ for ϕ≤t<ϕ+τ. Figure 3E shows a wave generated in this way. Figure 3F demonstrates that Cosinor is the best performing method on the dataset consisting of 500 non-oscillatory waves generated like in Fig. 3D and 500 symmetric non-stationary oscillatory waves generated like in Fig. 3E. While ODeGP has a poorer AUC value of 0.871 compared to 0.965 for Cosinor and 0.937 for eJTK, it still remains within the top three performing methods.

Asymmetric non-stationary oscillatory waves (like shown in Fig. 3H) were generated using a similar principle, but with a sawtooth waveform instead of a sine waveform. At the start of each new oscillation, a time period τ is sampled from $U(\tau_{1},\tau_{2})$. If the total time elapsed up to the start of this new oscillation is ϕ, then $f(t)=A\cdot \{\frac{t-\phi }{\tau }\}+N(0,\sigma )$ for ϕ≤t<ϕ+τ, where {} represents the fractional part function. RAIN can be seen as the best performing method in Fig. 3I for the dataset consisting of 500 non-oscillatory waves generated like in Fig. 3G and 500 asymmetric non-stationary oscillatory waves generated like in Fig. 3H. ODeGP however remains among the top three methods here as well.

A comparison of the performance of the methods tested across all simulated non-stationary datasets is shown in Table 3 (AUC values from all datasets are provided in Supplementary Section S5). ODeGP is the best performer for symmetric non-stationary datasets, being among the top three methods (in terms of AUC values) for all 15 datasets tested. In the non-stationary case as well, RAIN again emerges as the best method for asymmetric datasets. ODeGP shows a slight improvement in its relative performance on non-stationary datasets compared with stationary asymmetric datasets, being among the best three methods for a larger fraction of the datasets, and also never falling among the worst three methods.

**Table 3.** Comparison of the number of times each method tested appears among the best and worst performing three methods, in all non-stationary datasets considered.^a^

Non-stationary datasets				
Method	Symmetric datasets (total: 15)		Asymmetric datasets (total: 12)	
	Times among best three methods	Times among worst three methods	Times among best three methods	Times among worst three methods
Cosinor	9	2	4	5
eJTK	7	4	4	6
GPrank	2	4	0	6
JTK_Cycle	4	2	8	0
LSP	0	14	0	8
MetaCycle	2	6	5	1
ODeGP	15	0	5	0
SM Kernel	4	7	0	10
RAIN	2	6	10	0

a LSP, Lomb–Scargle periodogram; SM, spectral mixture.

### 3.4 ODeGP provides increased sensitivity at distinguishing oscillatory versus non-oscillatory patterns in noisy qPCR datasets

After analyzing simulated datasets, we next evaluated ODeGP’s ability to distinguish oscillatory and non-oscillatory patterns in experimental datasets and benchmarked its performance against existing methods. We started with a published dataset on primary mouse marrow stromal cells, where the expression levels of a number of circadian clock genes were measured over 48 h using qPCR (So *et al.* 2009). The cells were either treated with dexamethasone (Dex), which is expected to synchronize or stimulate clock gene expression oscillations, or with vehicle (DMSO) where weak or no oscillations are expected. Three independent experiments were done at each time point, thereby providing error bars for the expression levels as well. This dataset, therefore, represented a good test case for applying our rhythm detection method, since the ground truth is known.

**Figure 4.**
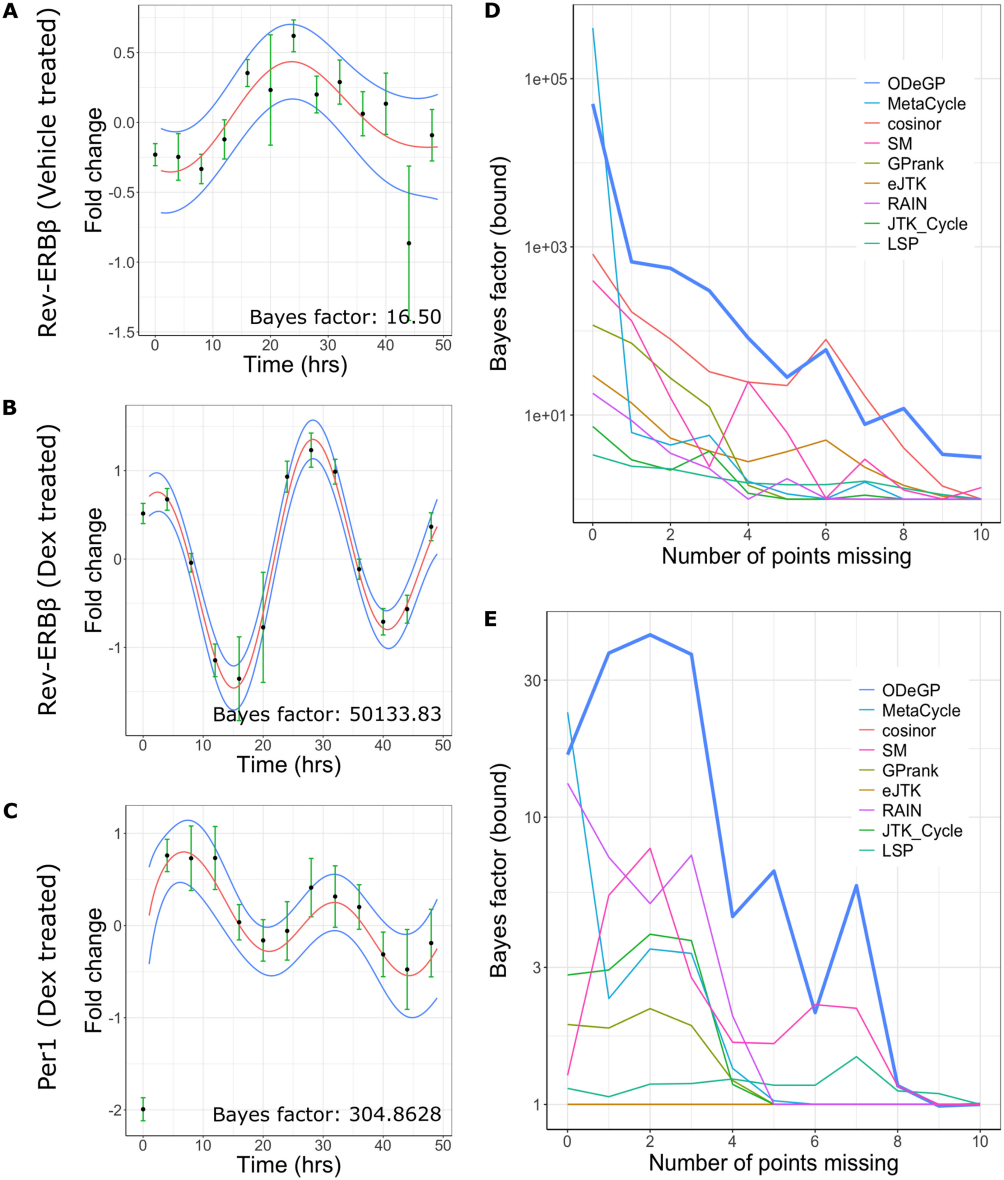
Application of ODeGP on *Rev-ERB*β and *Per1* expression data (So *et al.* 2009) demonstrates the sensitivity of ODeGP at distinguishing oscillatory from non-oscillatory data. (A) The relative gene expression of *Rev-ERB*β when treated with DMSO, measured at 4-h intervals over a 48-h duration, is shown by the black points with green error bars indicating an average taken over three biological replicates. Defining a GP prior using the non-stationary kernel and performing GP regression on this data produces the posterior distribution shown (mean in red, standard deviation in blue). (B) Relative gene expression of *Rev-ERB*β when treated with Dex, representing an oscillatory dataset, is shown by the black points with green error bars. The GP posterior of the non-stationary kernel applied to this data is shown with the posterior mean and standard deviation in red and blue, respectively. (C) Relative gene expression of *Per1*, when treated with Dex, is shown by the black points with green error bars. This dataset represents ground-truth oscillatory data, but with smaller amplitude oscillations as compared to (B). The GP posterior of the non-stationary kernel applied to this data is shown with the posterior mean and standard deviation in red and blue, respectively. ODeGP classifies this as oscillatory with much more confidence than other existing methods (see main text and Table 4). (D) Points from the raw data in (B) were removed one by one in a random order, and all methods were applied to the resulting down-sampled data at each step. The variation of the resulting Bayes factor (bounds) with increasing number of missing points is shown for each method. The thick blue line represents the ODeGP Bayes factor. (E) Analysis similar to panel (D) but for the raw data in panel (A).

The results from our analysis of the *Rev-ERB*β and *Per1* genes are shown in Fig. 4 (other genes are shown in Supplementary Fig. S2 and Supplementary Section S6). Raw data for *Rev-ERB*β, which exhibited large amplitude oscillations upon Dex synchronization, are shown in Fig. 4A and B along with the GP posteriors (mean and standard deviation) generated using the non-stationary kernel. Besides an AUC of its ROC curve being close to one, an additional characteristic of a good binary classifier is its ability to produce an output metric that is well separated for the two classes in consideration—in our case, non-oscillatory and oscillatory. Applying ODeGP on the vehicle treated data (Fig. 4A) produces a Bayes factor of 16.50, whereas it produces a Bayes factor of 50133.83 on the synchronized data in Fig. 4B, a separation of more than three orders of magnitude. The significant separation between these two values shows that ODeGP is able to make a clear distinction between the weakly oscillatory (see more in Section 4 about classifying oscillations based on Bayes factors) and strongly oscillatory qPCR data. The same trend is observed in the other genes we analyzed (Supplementary Fig. S2), where there is at least an order of magnitude increase in the Bayes factor, usually even more, when the data are oscillatory.

We next asked if ODeGP is more sensitive at detecting oscillations compared to other existing methods. To evaluate this, we analyzed *Per1* data from the same paper (So *et al.* 2009), which visibly exhibited lower amplitude oscillations with larger error bars (Fig. 4C). To compare the *P*-(or *Q*) values generated by other methods against the Bayes factor produced by ODeGP, we applied the conversion −1/(e p  log(p)) to the *P*-values to obtain corresponding Bayes factor bounds (Benjamin and Berger 2019) (Table 4). The Bayes factor bound represents an upper bound on Bayes factors corresponding to a given *P*-value *p*, under very general assumptions on the alternative hypothesis (Benjamin and Berger 2019). While the original derivation of this bound required the more stringent assumption of beta distributed *P*-values for the alternative hypothesis, later work extended it to a much less stringent requirement of a decreasing failure rate for the distribution of −log(p) (Sellke *et al.* 2001). The conversion used holds when p≤1/e; for larger values of *p*, the corresponding Bayes factor bound is taken as one.

**Table 4.** Comparison of output metrics of all methods tested on the low-amplitude *Per1* oscillation data shown in Fig. 4C, in descending order of the calculated Bayes factor (bound).^a^

Method	Metric type	Metric value	Bayes factor (bound)
ODeGP	Bayes factor	304.8628	304.8628
RAIN	*P*-value	0.00617	11.7191
eJTK	*P*-value	0.03577	3.0879
MetaCycle	*P*-value	0.04496	2.6376
Cosinor	*Q*-value	0.08537	1.7512
JTK_Cycle	*P*-value	0.12445	1.4185
SM Kernel	Bayes factor	1.2632	1.2632
LSP	*P*-value	0.43067	1.0140
GPrank	log of Bayes factor	−6.7185e-05	0.9999

a *P*-values/*Q*-values were converted to corresponding Bayes factor bound (Benjamin and Berger 2019) values where applicable to allow comparison with the Bayes factor returned by ODeGP. While eJTK, MetaCycle, and RAIN are the only other methods that correctly classify the dataset as oscillatory (based on a *P*-value cutoff of 0.05), the large difference between ODeGP’s Bayes factor and the upper bounds generated by these methods suggest that ODeGP is more sensitive at detecting oscillations.

As is clear from Table 4, the only methods besides ODeGP that correctly classified the data as rhythmic were eJTK, MetaCycle, and RAIN *(P-*values <0.05). However, the Bayes factor generated by ODeGP was much larger compared to the upper bound values of eJTK, Metacycle, or RAIN, demonstrating that ODeGP correctly classified the oscillations with much more confidence. Indeed, if recent guidelines for rejecting the null based on *P*-values <0.005 (Benjamin and Berger 2019) (see Section 4) were to be used, ODeGP would be the only method to correctly classify this dataset as oscillatory. The data’s oscillatory trend was also captured well in ODeGP’s non-stationary kernel posterior shown in Fig. 4C.

To more systematically test ODeGP’s sensitivity of detecting oscillations against existing methods, we compared the Bayes factor generated by ODeGP with the Bayes factor bound values produced by the other methods across an increasingly down-sampled dataset. The raw data for Rev-ERBβ in Dex-treated cells (Fig. 4B), which showed large amplitude oscillations, was considered as a starting point. Points were then removed from this dataset one by one in a random order, and at each step, all methods were applied to the sub-sampled data. Figure 4D demonstrates the rapid decrease in the Bayes factor and Bayes factor bounds with increasing missing points. At zero points missing all methods perform well (i.e. produce large Bayes factor bound values), though ODeGP and MetaCycle are distinctly better. As the number of missing points increases, the ODeGP Bayes factor (blue thick line in Fig. 4D) most consistently maintains a larger value compared to the Bayes factor bounds obtained from other methods. ODeGP thus performs better at identifying the data as oscillatory at an extent of missing points that causes other methods to fail. We also performed a similar downsampling analysis for the weakly oscillatory dataset in Fig. 4A, the results of which are shown in Fig. 4E. On comparing Fig. 4D and E, it is evident that for most points on the *x*-axis, the strong versus weak oscillation Bayes factors are best separated for ODeGP.

### 3.5 Cell-density dependent rapid emergence of oscillations in mESCs

**Figure 5.**
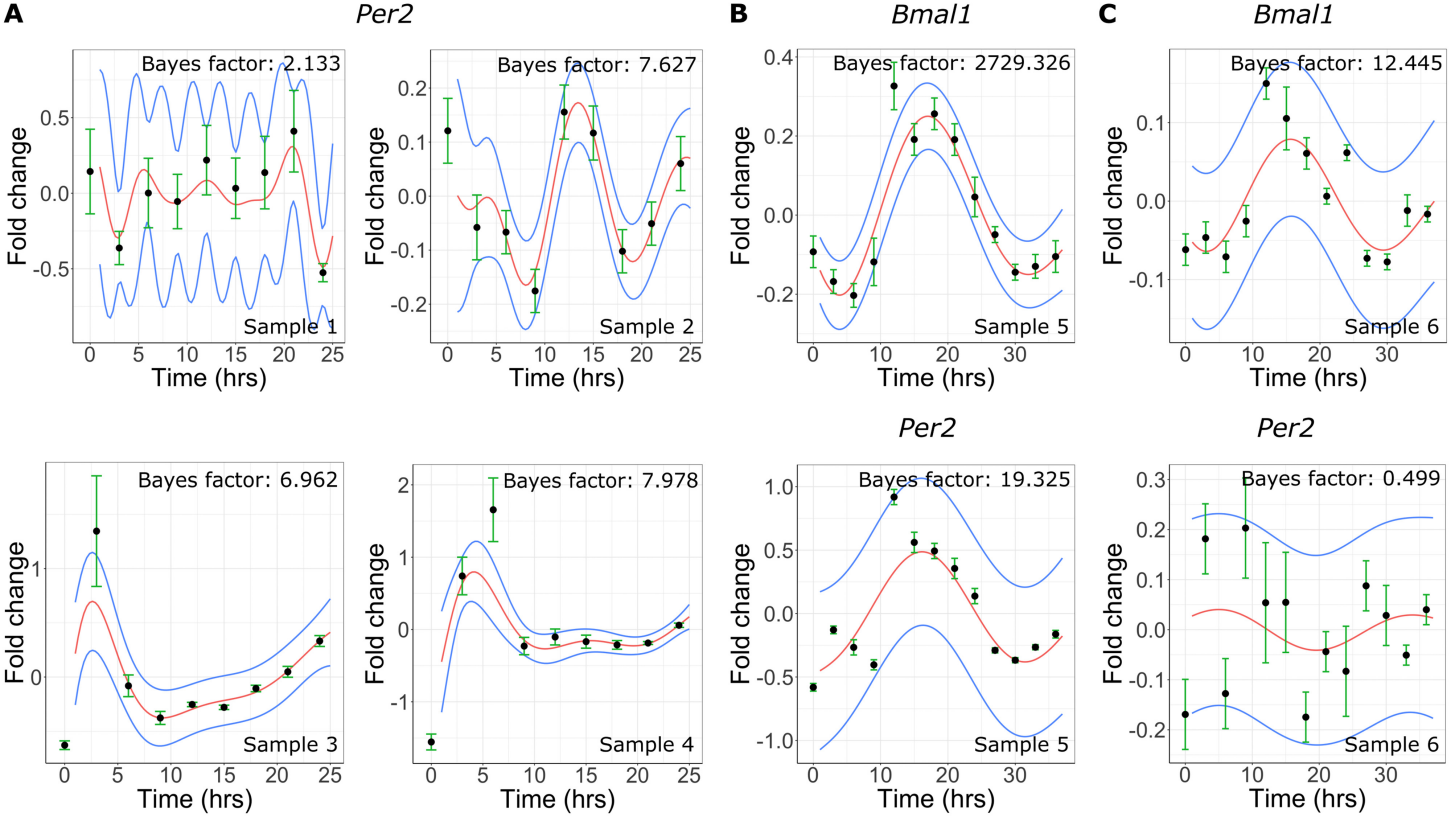
Cell-density dependent rapid emergence of oscillations in mESCs. GP posteriors of the non-stationary kernel (mean in red, standard deviation in blue) for (A) *Per2* gene expression from low-density samples 1, 2, 3, and 4 (see detailed descriptions in the Supplementary Material), (B) *Bmal1* and *Per2* expression for high-density sample 5, and (C) *Bmal1* and *Per2* expression for high-density (but 2i treated) sample 6. In all plots, black circles represent the mean qPCR measurement from three technical replicates.

Finally, we tested ODeGP’s ability to quantify oscillatory behavior in new qPCR datasets, that could enable novel biological discoveries. For this purpose, we used early passage pluripotent mESCs. Previous work has demonstrated that pluripotent stem cells do not exhibit oscillations of the core circadian clock genes, even though the genes are expressed in these cells (Yagita *et al.* 2010, Umemura and Yagita 2020). We first confirmed these well-established results by culturing mESCs on a variety of substrates in the presence of LIF where pluripotency is expected to be maintained (gelatin on plastic, fibronectin on glass, and laminin on glass; details in the Supplementary Section S1). We synchronized cells with Dex and collected the cells over a period of 24 h at intervals of 3 h. Consistent with the previous literature (Yagita *et al.* 2010, Umemura and Yagita 2020), application of our algorithm confirmed that there were no oscillations in any of the tested conditions, since the Bayes factors were in the range 2–8 (Fig. 5A). As we had observed in the last section, in known cases where there are no oscillations, the Bayes factor tends to be of the order of 10 while the presence of real oscillations pushes up the Bayes factor by one or two orders of magnitude.

We next asked if increasing the cell density could lead to the generation of oscillations of circadian clock gene expression. Previous work has demonstrated that about 2 weeks of RA induced differentiation can induce oscillations in mESCs (Yagita *et al.* 2010, Umemura and Yagita 2020), but to the best of our knowledge, the kinetics of oscillation development upon cell-density increase has not been explored in this cell type. Since higher cell density can potentially cause some amount of differentiation in stem cells (Wu *et al.* 2015), we explored the consequences of doubling the number of cells in our culture dish before Trizol extraction and gene expression quantification. We also extended the time over which cells were collected from 24 to 36 h, to allow for better detection of potential oscillations. Interestingly, though the higher cell density was maintained for only about 3–4 days (see Supplementary Section S1 for details), we saw clear signs of oscillation in *Bmal1* (Bayes factor of 2729) and weak oscillations in *Per2* (Bayes factor of 19), as can be seen in Fig. 5B. This was in contrast to the earlier studies using RA, where oscillations emerged only after 2 weeks (Yagita *et al.* 2010). To check whether cell differentiation could potentially have played a role in generation of these oscillations, we added the dual inhibitors of MEK/ERK and GSK3b (commonly called 2i) to an otherwise identical experimental set up with the higher cell density. 2i has been shown to differentially kill cells with low *Nanog* expression levels, which are prone to undergoing differentiation (Hastreiter *et al.* 2018). This time we found much lower Bayes factors for both *Bmal1* as well as *Per2*—12.4 and 0.5, respectively (Fig. 5C), clearly indicating that the oscillations are no longer present. These results suggest that increasing cell density might accelerate the development of oscillations of the core clock genes via some degree of differentiation, though the kinetics seem to be faster than that of RA-induced differentiation. Since differentiation is expected to directly increase the single cell oscillator amplitudes (Yagita *et al.* 2010) while increasing cell density is expected to increase coupling between the single cells (Noguchi *et al.* 2013, Finger *et al.* 2021), we also checked via mathematical modeling whether ODeGP is sensitive to both these forms of oscillation enhancement (Supplementary Section S9). Using a Poincare model for coupled oscillators, we found that for noisy single cell oscillators, increasing either the amplitude or the coupling strength leads to rapid increase in the ODeGP generated Bayes factors. If however the individual oscillators are less noisy or have high amplitudes, the Bayes factors are already large and do not increase further with increase in amplitude or coupling strength (Supplementary Fig. S4). Our modeling thus suggests that ODeGP is most sensitive to either routes of oscillation strengthening when the single cell oscillators are low-amplitude and noisy (Supplementary Fig. S4), as is expected in the stem cells. It is interesting to note that in some cases it is impossible to visually discern whether or not an oscillation is present (e.g. in Fig. 5A). These examples serve to highlight the importance of a quantitative and methodical approach to the oscillation-detection problem.

## 4 Discussion

Detecting biological oscillations from time-series datasets remains a challenging task even after decades of research. Here, we developed an oscillation-detection method based on GP regression for learning noisy patterns, combined with Bayesian model selection to distinguish between oscillatory and non-oscillatory datasets. Our method, ODeGP, is designed particularly to model non-stationarity using recently introduced kernels (Heinonen *et al.* 2015, Remes *et al.* 2017), as well as to model both the alternative (oscillatory) and null (non-oscillatory) hypotheses to avoid limitations of *P*-value-based algorithms. These advances set ODeGP apart from previous algorithms, including GP-based approaches to oscillation detection (a comparison with other GP-based methods is given in Supplementary Section S8). We demonstrated the improved performance of ODeGP compared to eight existing methods using both artificial (simulated) as well as previously published experimental datasets. In summary, our analysis broadly suggests that: (i) for symmetric waveforms, ODeGP almost always outperforms the other methods in detecting oscillations, both in stationary as well as non-stationary time-series data, and (ii) when oscillations have low amplitude, large error bars or missing data, ODeGP is more sensitive at detecting the rhythms—usually producing Bayes factors that are larger than those produced by other methods.

Applied to new qPCR data, we generated on circadian clock gene expression in pluripotent mESCs, ODeGP identified the development of oscillations in *Bmal1* and to some extent *Per2*, upon increasing cell density. While clock gene oscillations are known to be absent in both mouse (Yagita *et al.* 2010) and human (Dierickx *et al.* 2017) pluripotent embryonic stem cells, they have been shown to develop on a time scale of about 2 weeks on directed differentiation using RA (Yagita *et al.* 2010). Our results however suggest that the oscillations can be induced much faster, within 3–4 days, by increasing cell density (Fig. 5B). Interestingly, we found that these oscillations were prevented from developing in the presence of the inhibitors commonly known as 2i (Fig. 5C), thereby suggesting a potential role of density-dependent mESC differentiation in the establishment of the oscillations. Our current study is however limited by the fact that we did not vary cell density across a range of values, and neither did we measure the density at the time of RNA extraction. Future studies will be required to validate these early results and further explore the possibility that the kinetics of oscillation development depends on cell–cell signaling strength during stem–cell differentiation. These results may also have potentially interesting connections to recent experiments demonstrating cell-density effects on strengthening of the clock oscillations in differentiated cell types (Noguchi *et al.* 2013, Finger *et al.* 2021).

The oscillatory (alternate hypothesis) versus non-oscillatory (null) classification problem will necessarily involve defining somewhat arbitrary cutoffs. However, based on recent discussions on *P*-values and interpretation of Bayes factors as odds ratios, it has been proposed that a *P*-value of 0.005 is a more sensible cutoff compared to the widely used 0.05 value, corresponding to a Bayes factor bound of ∼14 (Benjamin and Berger 2019). We empirically notice that this guideline seems to be approximately consistent with our own experimental observations. In our case, the real “gold standard” non-oscillatory datasets are the pluripotent mESC datasets (Fig. 5A), where we expect no oscillations to be present even at the single cell level. In these datasets, we consistently find that the Bayes factors produced by ODeGP are below 14. The interpretation of the unsynchronized (vehicle treated), non-mESC datasets, such as in Fig. 4A, is more challenging. While these datasets are supposed to be non-oscillatory, we observe Bayes factors that are somewhat higher (16.5 in Fig. 4A and as high as 274 in Supplementary Fig. S2B). This suggests the presence of weak oscillations, which might be arising from plating of cells and/or addition of fresh media, which are both known to induce a small degree of synchronization between the single cell oscillators. After Dex synchronization however, the oscillations are expected to be stronger, which is correctly being reflected in each case by the much higher Bayes factors (Fig. 4B and C). Overall, consistent with the recent recommendations in the statistics community, our results suggest that a Bayes factor cutoff of 14 might be a good choice for classifying oscillatory versus non-oscillatory datasets. In addition, Bayes factors close to 14 could be classified as weak oscillations.

While our oscillation-detection method seems to significantly improve upon currently used methods, there are a number of limitations and potential areas of improvement. As can be seen from Tables 2 and 3, ODeGP is clearly inferior to RAIN in detecting oscillations in asymmetric waveforms. Providing ODeGP an enhanced ability to model asymmetric oscillations represents a clear avenue for improvement, which may be done by identifying new suitable functional forms for the hyperparameters of the non-stationary kernel. The runtime of our method is also significantly higher than that of most of the existing methods we tested (though it is comparable to that of RAIN), due to two main reasons—(i) the matrix inversion step associated with GPs and (ii) the hyperparameter optimization routine we used. Future work could improve on these aspects by use of structured-GPs (e.g. assuming a Markov property for the GP) allowing scalable inference (Liu *et al.* 2020) and using gradient-based methods for optimization (Heinonen *et al.* 2016). A further limitation of ODeGP is that the multiple-hypothesis correction provided is similar to the Bonferroni, rather than the Benjamini–Hochberg (BH) type correction in the frequentist setting (Westfall *et al.* 1997). The Bonferroni approach results in many false negatives when there are many hypotheses to be tested, and therefore ODeGP can currently be used only with smaller datasets, such as those generated in qPCR, eclosion, egg-laying, or feeding experiments. Detecting rhythmic or differentially rhythmic genes from genome-wide datasets using ODeGP could become possible in future with the development of BH-type corrections in the Bayesian setting.

## 5 Conclusions

While much work has been done in developing algorithms to detect oscillations in time-series datasets, there is clearly room for significant improvement. Our method combining GPs and Bayesian model selection demonstrates the flexibility of GPs, providing a highly sensitive approach for classifying oscillatory versus non-oscillatory datasets without using *P*-values. We hope that our results along with the user-friendly ODeGP R package, will spur more careful exploration of these approaches in the future. Applied to new experimental data, ODeGP provides initial evidence for rapid development of circadian clock oscillations upon increasing density of mESCs, and it would be exciting to see in future if these results have broader implications in the context of development and inter-cellular signaling.

## Supplementary Material

btad602_Supplementary_Data

## Data Availability

The data underlying this article will be shared on reasonable request to the corresponding author.
