# Supplementary material for: An improved rhythmicity analysis method using Gaussian Processes detects cell-density dependent circadian oscillations in stem cells
Source: Bioinformatics. 2023 Sep 28;39(10):btad602. doi: 10.1093/bioinformatics/btad602 (PMC10576164; doi:10.1093/bioinformatics/btad602)
Supplement: btad602_Supplementary_Data [file btad602_supplementary_data.pdf]

Supplementary Information for "An improved rhythmicity analysis method using Gaussian Processes detects cell-density dependent circadian oscillations in stem cells"

Shabnam Sahay[1,2], Shishir Adhikari[3,4], Sahand Hormoz[3,4,5*] and Shaon Chakrabarti[2,*]

[1]Department of Computer Science, Indian Institute of Technology Bombay
[2]Simons Centre for the Study of Living Machines, National Centre for Biological Sciences, Bangalore
[3]Department of Systems Biology, Harvard Medical School, Boston
[4]Department of Data Science, Dana-Farber Cancer Institute, Boston
[5]Broad Institute of MIT and Harvard, Cambridge

Contents

1 qPCR data collection protocol **1**

2 Fold-change and error analysis for qPCR datasets **3**

3 An overview of Gaussian Process Regression **4**

4 Implementation details of ODeGP **5**

5 Evaluation against existing methods **9**

6 Analysis of model complexity penalisation by MLL **13**

7 Application of ODeGP to qPCR datasets **14**

8 Comparison of ODeGP with other GP based algorithms **16**

9 Modeling coupled oscillators to investigate the role of amplitude and coupling strength on the Bayes Factor **16**

1 qPCR data collection protocol

For the qPCR experiments to investigate the presence/absence of oscillations, we used the following protocol for Samples 1-4 (a schematic is presented in Figure 1). A frozen vial of mESCs was first thawed and grown on a 100 mm plastic dish coated with gelatin, under pluripotent conditions, till about 60-70% confluency. The cells were then split and grown on a six well plate for about a week (with passaging) under the conditions mentioned in Table 1. Finally, for Dex synchronization and Trizol collection, the cells were transferred to 24 well-plates under the same growth conditions as mentioned in Table 1. To ensure equal cell density at time of Trizol collection, during transfer to the 24-well plates two groups were created - “Day 1” cells were plated into 5 wells of a 24-well plate corresponding to eventual collection time-points 12, 15, 18, 21 and 24 hours, while “Day 2” cells were plated on 4 wells of a separate 24-well plate representing eventual collection time-points 0, 3, 6 and 9 hours (Figure 1). Thus one full 24-hour cycle with 3-hour sampling was represented. For the Day 1 cells, 25000 cells were seeded per well while for the Day 2 cells, 12500 cells were seeded per well. After allowing a few hours for attachment in the 24 well plates, the Day 1 cells were Dex synchronized beginning from 9 am in the following manner: time-point 24 synchronized at 9am, time-point 21 at 12pm, all the way till time-point 12 which was synchronized at 9 pm. Finally, these Day 1 cells were Trizol collected next day at 10 am. Two hours later, beginning from 12 pm, the Day 2 cells were synchronized every three hours, and finally collected using Trizol at 10 pm (Figure 1). This somewhat elaborate strategy was followed to ensure that at time of collection, all time points had similar cell densities. Assuming an average division time of 12 hours for mESCs, this protocol allowed for 2 doubling times for the Day 1 cells and 3 doubling times for the Day 2 cells. The final expected cell numbers at time of Trizol collection are shown in Table 1. For Samples 5-6, an identical protocol was followed, but with an extra day and an additional collection time, to allow sample RNA collection over 36 hours. Samples 1-4 are considered “low density” since at collection they were about 40-60% confluent while Samples 5-6 are considered “high density” since they were almost completely confluent at time of collection. Note that this high density was achieved only within the 2-3 days that the cells were plated in the 24-well plates, since prior to that the cells

were always maintained at less than 60-70% confluency in the 100 mm dish or 6-well plate.

**Sample number**	**Growth conditions**	**Seeding density**	**Expected cell number at time of Trizol collection**
1; 24 hrs	Gelatin on plastic, media with LIF	Day 1 cells - 25000 Day 2 cells - 12500	100000 ("low density")
2; 24 hrs	Fibronectin on glass, media with LIF	Day 1 cells - 25000 Day 2 cells - 12500	100000 ("low density")
3; 24 hrs	Fibronectin on glass, media with LIF	Day 1 cells - 40000 Day 2 cells - 20000	160000 ("low density")
4; 24 hrs	Laminin on glass, media with LIF	Day 1 cells - 40000 Day 2 cells - 20000	160000 ("low density")
5; 36 hrs	Laminin on glass, media with LIF	Day 0 cells - 20000 Day 1 cells - 10000 Day 2 cells - 5000	320000 ("high density")
6; 36 hrs	Laminin on glass, media with LIF + 2i	Day 0 cells - 20000 Day 1 cells - 10000 Day 2 cells - 5000	$\sim$ 320000 ("high density")

Table 1: Description of various samples, seeding densities and substrates used for the qPCR experiments.

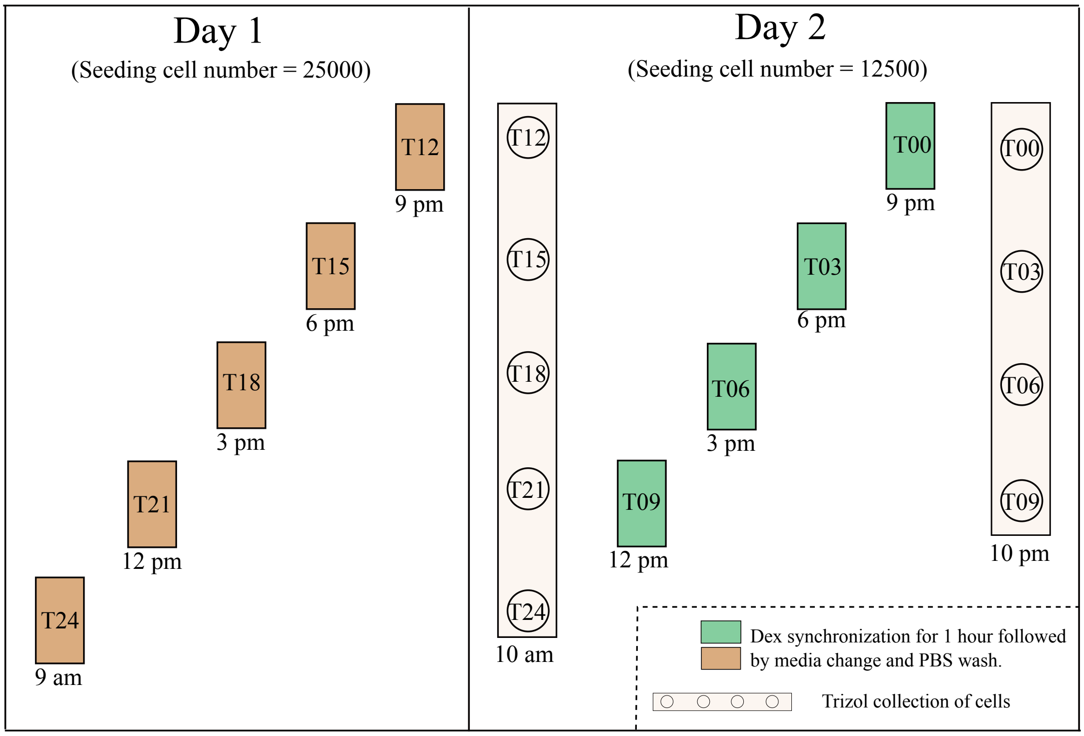

Figure 1: Schematic of qPCR experimental design to maintain the same cell density across all time points. Each rectangular block (brown and green representing Day 1 and Day 2 cells respectively) corresponds to a sample that was dexamethasone synchronized for 1 hour. Times of dex-synchronization are mentioned below each block. Trizol collection was performed at 10 am on Day 2 for Day 1 cells, and at 10 pm on Day 2 for Day 2 cells. The circadian time (time after synchronization) is mentioned inside each block. This protocol ensured that at the time of RNA collection, each sample across the 24 hours had approximately same cell density.

2 Fold-change and error analysis for qPCR datasets

Fold change computation

We collected Ct values for a gene of interest from three technical replicates of each sample. For every sample, we also collected three Ct values for the reference gene (i.e. GAPDH). The calculation for fold change is as follows:

- We take the average for three samples ($\langle C_s \rangle$) and three reference samples($\langle C_r \rangle$):

$$\langle C_s \rangle = \frac{1}{3} \sum_{i=1}^{3} C_s^{(i)} \tag{1}$$

$$\langle C_r \rangle = \frac{1}{3} \sum_{i=1}^{3} C_r^{(i)} \tag{2}$$

- We calculate the difference of these two: $\Delta \text{ct} = \langle C_s \rangle - \langle C_r \rangle$.
- Δct at time point zero is used as reference Δct. We named it $\Delta \text{ct}_{\text{Ref}}$.
- We calculate $\Delta\Delta$ct as the difference between Δct and $\Delta \text{ct}_{\text{Ref}}$.
- The fold change is defined as $2^{-\Delta\Delta \text{ct}}$

Error propagation

To calculate the error in fold change, we did basic error propagation computation as follows:

- Uncertainty in fold change depends on the error in $\Delta\Delta$ct as follows:

$$\delta_{fc} = \delta_{fc/\Delta\Delta\text{ct}} = \left| \frac{\partial 2^{-\Delta\Delta\text{ct}}}{\partial \Delta\Delta\text{ct}} \right| \delta_{\Delta\Delta\text{ct}} \tag{3}$$

  where, $\delta_{fc/\Delta\Delta\text{ct}}$ is uncertainty in fold change due to uncertainty in $\Delta\Delta$ct and $\delta_{\Delta\Delta\text{ct}}$ is uncertainty in $\Delta\Delta$ct

- $\delta_{\Delta\Delta\text{ct}}$ depends on uncertainty in $\Delta\Delta$ct due to uncertainty in Δct ($\delta_{\Delta\Delta\text{ct}/\Delta\text{ct}}$) and uncertainty in $\Delta\Delta$ct due to uncertainty in $\Delta\text{ct}_{\text{Ref}}$ ($\delta_{\Delta\Delta\text{ct}/\Delta\text{ct}_{\text{Ref}}}$) as follows:

$$\delta_{\Delta\Delta\text{ct}} = \sqrt{(\delta_{\Delta\Delta\text{ct}/\Delta\text{ct}})^2 + (\delta_{\Delta\Delta\text{ct}/\Delta\text{ct}_{\text{Ref}}})^2} \tag{4}$$

- $\delta_{\Delta\Delta\text{ct}/\Delta\text{ct}}$ can be calculated as follows:

$$\delta_{\Delta\Delta\text{ct}/\Delta\text{ct}} = \left| \frac{\partial(\Delta\Delta\text{ct})}{\partial \Delta\text{ct}} \right| \delta_{\Delta\text{ct}} = -\Delta\text{ct}_{\text{Ref}} \times \delta_{\Delta\text{ct}} \tag{5}$$

  where $\Delta\Delta\text{ct} = \Delta\text{ct} - \Delta\text{ct}_{\text{Ref}}$ and $\delta_{\Delta\text{ct}}$ is the uncertainty in Δct

- Similarly, $\delta_{\Delta\Delta\text{ct}/\Delta\text{ct}_{\text{Ref}}}$ can be calculated as follows:

$$\delta_{\Delta\Delta\text{ct}/\Delta\text{ct}_{\text{Ref}}} = \left| \frac{\partial(\Delta\Delta\text{ct})}{\partial \Delta\text{ct}_{\text{Ref}}} \right| \delta_{\Delta\text{ct}_{\text{Ref}}} = \Delta\text{ct} \times \delta_{\Delta\text{ct}_{\text{Ref}}} \tag{6}$$

  where $\delta_{\Delta\text{ct}_{\text{Ref}}}$ is the uncertainty in $\Delta\text{ct}_{\text{Ref}}$

- $\delta_{\Delta\text{ct}}$ can be calculated as follows:

$$\delta_{\Delta\text{ct}} = \sqrt{(\delta_{\Delta\text{ct}/\langle C_s\rangle})^2 + (\delta_{\Delta\text{ct}/\langle C_r\rangle})^2} \tag{7}$$

- $\delta_{\Delta\text{ct}_{\text{Ref}}}$ is just the special case of equation 7, calculated at time point zero.
- $\delta_{\Delta\text{ct}/\langle C_s\rangle}$ can be calculated as follows:

$$\delta_{\Delta\text{ct}/\langle C_s\rangle} = \left|\frac{\partial\Delta\text{ct}}{\partial\langle C_s\rangle}\right| \delta_{\langle C_s\rangle} = -\langle C_r\rangle \times \delta_{\langle C_s\rangle} \tag{8}$$

  where $\delta_{\langle C_s\rangle}$ standard error of mean for $C_s(i)$

- $\delta_{\Delta\text{ct}/\langle C_r\rangle}$ can be calculated as follows:

$$\delta_{\Delta\text{ct}/\langle C_r\rangle} = \left|\frac{\partial\Delta\text{ct}}{\partial\langle C_r\rangle}\right| \delta_{\langle C_r\rangle} = -\langle C_s\rangle \times \delta_{\langle C_r\rangle} \tag{9}$$

  where $\delta_{\langle C_r\rangle}$ standard error of mean for $C_r(i)$

3 An overview of Gaussian Process Regression

Gaussian processes (GP) provide a framework for performing non-parametric regression to estimate a target function via Bayesian inference [1]. A function-space rather than weight-space approach is taken in GP regression, where instead of fixing a function and placing a prior on its parameters (to learn the optimal parameters via Bayesian inference), the functional form itself is learned by placing a GP prior over functions. A GP is simply a collection of random variables, any finite combination of which has a multivariate normal distribution. If each random variable represents the value assumed by the target function f at a particular domain point x, then the joint distribution of these random variables corresponds to the prior distribution over possible target functions. The multivariate normal is described by a covariance matrix Σ, whose entries are calculated from a kernel function capturing the smoothness and length-scales associated with the data. Note that the "parameters" of the kernel are actually hyperparameters of the inference problem – they do not directly specify any one functional form to be learnt; rather they help prioritize a class of functions with certain smoothness properties and decay lengthscales.

Let X^* be the vector of domain points $x \in \mathbb{R}$ at which the value of f is to be estimated. Then the prior distribution of $f(X^*)$ (before any data is observed) is given by

$$f(X^*) \sim \mathcal{N}(\mu(X^*), \Sigma_{X^*X^*}) \tag{10}$$

Here, $\mu : \mathbb{R} \to \mathbb{R}$ is a 'mean' function such that $\mu(X^*) = \mathbb{E}[f(X^*)]$. The covariance matrix $\Sigma_{X^*X^*}$ is determined by a positive semi-definite kernel function $K : \mathbb{R}^2 \to \mathbb{R}$ as follows:

$$\Sigma_{ij} = Cov(f(x_i), f(x_j)) = K(x_i, x_j) \text{ for } x_i, x_j \in X^* \tag{11}$$

K thus describes the similarity between values of f at different domain points. The parameters of K are considered to be hyperparameters in the context of the regression. With μ and K being well-defined on $\mathbb{R}$ and $\mathbb{R}^2$ respectively, X^* can contain any number of $x \in \mathbb{R}$, allowing us to model f at a theoretically infinite number of domain points.

Given a vector of N observed data values Y at corresponding domain points X, Bayesian inference can be carried out to obtain a posterior distribution for f:

$$f(X^*|Y) \sim \mathcal{N}(\mu^*(X^*), \Sigma^*_{X^*X^*}) \tag{12}$$
$$\text{where } \mu^*(X^*) = \mu(X^*) + \Sigma_{X^*X}\Sigma^{-1}_{XX}(Y - \mu(X))$$
$$\text{and } \Sigma^*_{X^*X^*} = \Sigma_{X^*X^*} - \Sigma_{X^*X}\Sigma^{-1}_{XX}\Sigma_{XX^*}$$

An optimal posterior that best models the given data while avoiding overfitting can be found by maximization of the data likelihood $P(Y|f) = \mathcal{N}(Y|\mu, K)$. The log marginalized form of this likelihood can be expressed as

$$\log P(Y|\mu, K) = -\frac{1}{2}Y^T K^{-1}_{XX} Y - \frac{1}{2}\log|K_{XX}| - \frac{N}{2}\log(2\pi) \tag{13}$$

With the hyperparameters of K being the only variables here, this marginal log likelihood can be maximized by finding optimal values for the hyperparameters using standard techniques.

4 Implementation details of ODeGP

Pre-processing the time-series data

Let X be a list of N points in the domain at which the data has been observed. In our case, X is a list of times. If d distinct replicates of the data have been obtained for the N time points in X, let these be $Y_1, Y_2, \ldots, Y_d$. The average and standard deviation of these d lists at each of the N domain points is calculated to obtain Y and S respectively. This step is not carried out if Y and S are directly supplied as input to the method.

Next, Y is detrended. This is done by performing simple linear regression on the points in Y, and setting the new Y as the difference between the initial Y and the values of the resulting best-fit line at the corresponding time points in X.

Finally, Y is 0-centered by setting $Y = Y - mean(Y)$. This allows us to set the GP prior distribution's mean as zero, and simplifies further computations required for GP regression.

Kernel definitions

The diagonal kernel K_D and the non-stationary kernel K_{NS} are positive semi-definite functions $(R \times R) \rightarrow R$ defined as follows:

$$K_D(x, x') = \epsilon^2 \cdot \delta_{xx'} \tag{14}$$
$$\text{where } \delta_{xx'} = \begin{matrix} 0, & x \neq x' \\ 1, & x = x' \end{matrix}$$

$$K_{NS}(x, x') = \sum_{i=1}^{Q} w_i(x) w_i(x') k_{gibbs,i}(x, x') \cos\left(2\pi\left(\frac{x}{\mu_i(x)} - \frac{x'}{\mu_i(x')}\right)\right) + \epsilon_i^2 \cdot \delta_{xx'} \tag{15}$$

We use K_{NS} with $Q = 1$, resulting in the following updated expression:

$$K_{NS}(x,x') = w(x)w(x')k_{gibbs}(x,x')\cos\left(2\pi\left(\frac{x}{\mu(x)} - \frac{x'}{\mu(x')}\right)\right) + \epsilon^2 \cdot \delta_{xx'} \tag{16}$$

$$\text{where } k_{gibbs}(x,x') = \sqrt{\frac{2l(x)l(x')}{l(x)^2 + l(x')^2}}\exp\left(-\frac{(x-x')^2}{l(x)^2 + l(x')^2}\right), \text{ and}$$

$$w(x) = w_{max} - (w_{max} - w_{min})e^{-c_w t}$$
$$l(x) = l_{max} - (l_{max} - l_{min})e^{-c_l t}$$
$$\mu(x) = \mu_{max} - (\mu_{max} - \mu_{min})e^{-c_\mu t}$$

The diagonal kernel has a single hyperparameter ϵ, whereas the non-stationary kernel has ten hyperparameters (ϵ, along with three each for the given functional forms of $w(x), l(x)$ and $\mu(x)$). ϵ acts as a global noise term in these kernels. The local noise encoded by S is incorporated as follows: for $s_i \in S$, s_i^2 is added to the ith diagonal entry of the covariance matrix (irrespective of which kernel it is constructed with).

The optimization routine

Given the data, optimal hyperparameters for each of K_D and K_{NS} are found through maximization of the marginal log likelihood (MLL). This is the log likelihood of observing the data, given the kernel and the domain points at which the observations are made, i.e. $\log P(Y|K,X)$.

$$\begin{aligned} \text{MLL} &= \log P(Y|K,X) = \log \mathcal{N}(Y|0, K_{XX}) \\ &= -\frac{1}{2}Y^T K_{XX}^{-1} Y - \frac{1}{2}\log|K_{XX}| - \frac{N}{2}\log(2\pi) \end{aligned} \tag{17}$$

where K_{XX} is $K(X,X)$, i.e. the $N \times N$ covariance matrix generated by applying the kernel function K on all pairs of points (x_i, x_j) such that $x_i, x_j \in X$, and then adding s_i^2 to K_{ii} for $s_i \in S$.

The DEoptim [2] package, which uses a differential evolution algorithm, is used to carry out this optimization in ODeGP. The DEoptim method is designed to exclusively carry out minimizations, so the negative MLL is passed to this method as the function to be optimized.

The (negative) MLL, as seen from 17, is a function of X, Y and K. Since both X and Y are fixed by a given dataset, the optimization is done by searching over different possible K, or more specifically different sets of hyperparameters for K.

To carry out its optimization, DEoptim requires specification of the upper and lower bounds of the search space for each hyperparameter. For the purpose of defining these bounds, some measures are derived from X and Y:

- `X_range`: $max(X) - min(X)$
- `timestep`: $\frac{max(X)-min(X)}{length(X)+1}$
- `Y_range`: $max(Y) - min(Y)$
- `Y_step`: the maximum difference between two consecutive values in the list obtained by arranging the values in Y in ascending order

K_D has a single hyperparameter, ϵ. Since this encodes global noise in ODeGP, we pass the lower and upper bounds for this hyperparameter to DEoptim as 0 and `Y_range` respectively. The same bounds are used for the ϵ hyperparameter in K_{NS}.

The bounds for the remaining nine hyperparameters in K_{NS} are defined in Table 2:

Hyperparameter	Lower bound	Upper bound
w_{max}	`Y_step`	`Y_range`
w_{min}	0	`Y_step`
c_w	0	`X_range`
l_{max}	`timestep`	`X_range`
l_{min}	0	`timestep`
c_l	0	`X_range`
μ_{max}	`X_range/2`	`X_range`
μ_{min}	`timestep`	`X_range/2`
c_μ	0	`X_range`

Table 2: List of the hyperparameters and their lower and upper bounds used in optimization of the marginal log likelihood.

These hyperparameters control the variation of $w(x)$, $l(x)$ and $\mu(x)$ through the functional form described in Equation 16, which is introduced in [3]. Their bounds are set as above with the following reasoning:

- w_{min} and w_{max} are respectively the minimum and maximum possible value taken by $w(x)$, which controls the amplitude of the correlation between the GP posterior's values at different time points. Since `Y_range` is the amplitude of the data as a whole, we take $w_{max} \leq$ `Y_range`. w_{min} cannot exceed `Y_step`, else $w(x)$ will not be able to generate correlations with amplitudes comparable to the difference between closely spaced Y values. `Y_step` thus also acts as a lower bound for w_{max}.

- l_{min} and l_{max} are respectively the minimum and maximum possible value taken by $l(x)$, which encodes the lengthscale of correlation between the GP posterior values at different time points. It is clear that $0 \leq l(x) \leq$ `X_range`. l_{max} cannot be smaller than `timestep`, else $l(x)$ will not be able to generate correlations between consecutive time points. `timestep` thus also acts as an upper bound for l_{min}.

- μ_{min} and μ_{max} are respectively the minimum and maximum possible value taken by $\mu(x)$, which controls the GP posterior's oscillation period at different time points. $\mu_{min} \geq$ the sampling interval of the data (i.e. `timestep`). $\mu_{max} \leq$ the total time range of the data (`X_range`), since for time periods $>$ `X_range` a complete oscillation will not be present in the data. μ_{max} cannot be smaller than `X_range/2`, else larger time periods possibly present in the data will not be captured. `X_range/2` thus also acts as an upper bound for μ_{min}.

- c_w, c_l and c_μ control the 'curvature' of the functions $w(x)$, $l(x)$ and $\mu(x)$; i.e. the speed with which they respectively approach their respective maximum values [3]. All of these are bounded between 0 and `X_range`, with the assumption that this curvature should not exceed the total domain range of the data.

The minimization of negative MLL is done with a maximum of 50 iterations for K_D, and 200 iterations for K_{NS}. Further parameter values used that are specific to DEoptim's implementation can be found in the code for ODeGP.

When complete, the optimization returns the set of hyperparameter values h for the kernel in consideration that results in the minimum attained negative MLL value. Let this be h_D and h_{NS} for K_D and K_{NS} respectively.

Model selection with the Bayes factor

Given X and Y, h_D and h_{NS}, let the corresponding optimal MLL values for K_D and K_{NS} be MLL_D and MLL_{NS} respectively.

The Bayes factor is calculated as a ratio of the marginal likelihoods of two competing hypotheses. In our case, it compares the suitability of the two kernels (models) for the given data. Since the MLL values previously calculated are log marginal likelihoods, the Bayes factor is evaluated by applying the exponential function to the difference of MLL_{NS} and MLL_D.

$$\text{BayesF} = \exp\ (\text{MLL}_{NS} - \text{MLL}_D) \tag{18}$$

The calculated BayesF is returned by the method as output. The larger the resulting value of BayesF, the stronger the support for K_{NS} being a better model for the data. Hence, if this metric exceeds a defined threshold then the data is judged to be oscillatory, else it is not.

The posterior distribution and further processing

The GP posterior distribution for kernel K at any given list of M time points W has a mean μ and covariance matrix Σ which can now be calculated as follows:

$$\mu = K_{XW}^T K_{XX}^{-1} Y \tag{19}$$

$$\Sigma = K_{WW} - K_{XW}^T K_{XX}^{-1} K_{XW} \tag{20}$$

where K_{XW} is the $N \times M$ covariance matrix $K(X, W)$, K_{WW} is the $M \times M$ covariance matrix $K(W, W)$ and K_{XX} is the same as defined in 17. Here, while evaluating the kernel function, the hyperparameters of K are fixed as h, obtained through optimization of the MLL.

The confidence interval of μ at time point $w_i \in W$ is evaluated as $2\sqrt{\Sigma_{ii}}$. The strongest detected oscillation period in μ is obtained using R's `spectrum` function from the `stats` library.

Correction to the Bayes Factor for multiple-hypothesis testing

While ODeGP is primarily intended for detecting oscillations in single trajectories, a multiple hypothesis testing correction in the Bayesian setting is also provided in case a dataset has multiple trajectories that need to be simultaneously analyzed for the presence of rhythms. Here we provide a brief explanation of the correction factor, and how it compares with the frequentist Bonferroni approach of controlling the Family-Wise Error Rate.

As discussed above, ODeGP computes the Bayes factor as the ratio of the marginal likelihoods of the alternate (oscillatory; H_A) versus null (not-oscillatory; H_0) hypotheses. Using Bayes theorem, it is easy to show that:

$$\frac{\pi(H_A|Y)}{\pi(H_0|Y)} = \frac{\pi(H_A)}{\pi(H_0)} \frac{P(Y|H_A)}{P(Y|H_0)}, \tag{21}$$

where the left hand side represents the posterior odds, and the right hand side is the product of the prior odds and the Bayes Factor. When using just the Bayes factor to classify a waveform as oscillatory or not, ODeGP implicitly assumes that the prior odds is 1, i.e. $\pi(H_A) = \pi(H_0)$. This results in the posterior odds being identical to the Bayes factor. In this setting, multiple hypothesis corrections can be carried out by suitably modifying the prior odds to generate a posterior odds that is less than the Bayes factor [4], as described below.

Assume there are k trajectories that are to be tested for oscillations, with corresponding null and alternate hypotheses H_{0i}, H_{Ai} respectively, where $i = 1, ..., k$. Suppose that initially $\pi(H_{0i})$ is set to 0.5 for all i, implying that the assessor is truly objective about the prior probability of the hypotheses being true or false. Given independent null hypotheses, the joint probability of all the null hypotheses being true becomes 0.5^k, which is possibly much smaller than desired. In this case, one could begin with assigning a value for the joint probability $\Pi_0 = \prod_{i=1}^{k} \pi(H_{0i})$, for instance 0.5, and then modifying the $\pi(H_{0i})$ accordingly. If there is no preference for any particular H_{0i}, then the revised $\pi(H_{0i})$ becomes equal to $\Pi_0^{1/k}$. In this case, the prior odds for each trajectory is no longer 1 and the posterior odds in Equation. 21 gets modified to:

$$\frac{\pi(H_{Ai}|Y)}{\pi(H_{0i}|Y)} = \left(\frac{1 - \Pi_0^{1/k}}{\Pi_0^{1/k}} \right) \frac{P(Y|H_{Ai})}{P(Y|H_{0i})}. \tag{22}$$

Note that the Bayes factor for each hypothesis, $\frac{P(Y|H_{Ai})}{P(Y|H_{0i})}$, is calculated independently as explained previously. ODeGP reports this prior odds as a correction term to the Bayes factor, with a default value $\Pi_0 = 0.5$. It can be easily demonstrated (see [4] for the derivation) that under certain conditions ($k \to \infty$ and 1/Bayes factor $\to 0$), this method of multiple hypothesis correction is similar to the frequentist Bonferroni correction, since the modified posterior probability $\pi(H_{Ai}|Y)$ is scaled by a factor approximately equal to k.

5 Evaluation against existing methods

Generating simulated datasets

Simulated waves were generated as sets of 3 replicates, with a duration of 48 hours and an interval of 3 hours between consecutive observations (when no data was missing). Each such set of 3 replicates was produced as follows: a base waveform was generated at the designated timepoints - let these times be contained in a vector T, the corresponding waveform values be contained in a vector D, and the length of T = length of $D = N$. Taking the standard deviation of D to be σ_D, each replicate was generated by adding to D, a vector of N independent samples drawn from $U(-\sigma_D, \sigma_D)$.

The base waveform D is generated by applying a function $f(t)$ to the times in T (see Tables 3, 4, 5 and 6 for details). Following this, a fraction γ of the times in T are randomly chosen, and these times along with the corresponding values in D are deleted from T and D respectively.

Construction of an ROC curve requires a dataset consisting of both oscillatory and non-oscillatory waves. Each such dataset was generated as a collection of 500 oscillatory waves and 500 non-oscillatory waves, where each wave is a set of 3 replicates produced from a defined base waveform as described above.

For oscillatory waves, the definitions of f depend on the type of waves being simulated, and are provided in Tables 3, 4, 5 and 6 along with the values of γ used in different cases. For non-

oscillatory waveforms, f was defined as $f(t) = \mathcal{N}(0, \sigma)$, and $\gamma =$ the γ being used to generate the 500 oscillatory waves comprising the other half of that particular dataset of 1000.

**Stationary symmetric waves**	
$\mathbf{f(t)}$	$A_1 \sin(2\pi t/\tau_1) + A_2 \sin(2\pi t/\tau_2) + \mathcal{N}(0, \sigma)$
Dataset no.	Parameter values
1,2,3,4	$A_1 = 3, \tau_1 = 22, A_2 = 3, \tau_2 = 28$<br>$(\sigma, \gamma) = (0.1, 0.0)$ or $(0.1, 0.5)$ or $(1, 0.0)$ or $(1, 0.5)$
5,6,7,8	$A_1 = 3, \tau_1 = 18, A_2 = 3, \tau_2 = 26$<br>$(\sigma, \gamma) = (0.1, 0.0)$ or $(0.1, 0.5)$ or $(1, 0.0)$ or $(1, 0.5)$
9	$A_1 = 5, \tau_1 = 24, A_2 = 0, \tau_2 = 24$<br>$(\sigma, \gamma) = (1, 0.5)$

Table 3: Functional forms and parameter values used to generate simulated stationary symmetric datasets.

**Stationary asymmetric waves**	
$\mathbf{f(t)}$	$A \cdot \{t/\tau\} + \mathcal{N}(0, \sigma)$<br>where $\{\}$ represents the fractional part function
Dataset no.	Parameter values
10,11,12,13	$A = 5, \tau = 18$<br>$(\sigma, \gamma) = (0.1, 0.0)$ or $(0.1, 0.5)$ or $(1, 0.0)$ or $(1, 0.5)$
14,15,16,17	$A = 5, \tau = 26$<br>$(\sigma, \gamma) = (0.1, 0.0)$ or $(0.1, 0.5)$ or $(1, 0.0)$ or $(1, 0.5)$

Table 4: Functional forms and parameter values used to generate simulated stationary asymmetric datasets.

**Non-stationary symmetric waves**	
$\mathbf{f(t)}$ (type 1 - monotonically decreasing time period)	$A \cdot \sin(\frac{2\pi t}{1+\lvert t-\tau \rvert}) + \mathcal{N}(0, \sigma)$<br>where $\tau \geq 48$
Dataset no.	Parameter values
18,19,20,21	$A = 5, \tau = 72$<br>$(\sigma, \gamma) = (0.1, 0.0)$ or $(0.1, 0.5)$ or $(1, 0.0)$ or $(1, 0.5)$
22,23,24,25	$A = 5, \tau = 60$<br>$(\sigma, \gamma) = (0.1, 0.0)$ or $(0.1, 0.5)$ or $(1, 0.0)$ or $(1, 0.5)$
26,27,28,29	$A = 5, \tau = 48$<br>$(\sigma, \gamma) = (0.1, 0.0)$ or $(0.1, 0.5)$ or $(1, 0.0)$ or $(1, 0.5)$
$\mathbf{f(t)}$ (type 2 - randomly varying time period)	At $t = 0$ OR at the completion of each time period, a new time period τ is sampled from $\mathcal{N}(\mu, \sigma)$. If the total time elapsed upto the start of this new oscillation is ϕ, then $f(t) = A \cdot \sin(\frac{2\pi(t-\phi)}{\tau}) + \mathcal{N}(0, \sigma)$ for $\phi \leq t < \phi + \tau$
Dataset no.	Parameter values
30,31,32	$\mu = 24, \sigma = 1.33, A = 1.5$<br>$(\sigma, \gamma) = (1, 0.0)$ or $(1, 0.5)$ or $(0.5, 0.5)$

Table 5: Functional forms and parameter values used to generate simulated non-stationary symmetric datasets.

Non-stationary asymmetric waves	
$\mathbf{f(t)}$	At $t = 0$ OR at the completion of each time period, a new time period τ is sampled from $U(\tau_1, \tau_2)$. If the total time elapsed upto the start of this new oscillation is ϕ, then $f(t) = A \cdot \{\frac{t-\phi}{\tau}\} + \mathcal{N}(0, \sigma)$ for $\phi \leq t < \phi + \tau$, where $\{\}$ represents the fractional part function
Dataset no.	Parameter values
33,34,35,36	$\tau_1 = 12, \tau_2 = 30, A = 5$<br>$(\sigma, \gamma) = (0.1, 0.0)$ or $(0.1, 0.5)$ or $(1, 0.0)$ or $(1, 0.5)$
37,38,39,40	$\tau_1 = 12, \tau_2 = 21, A = 5$<br>$(\sigma, \gamma) = (0.1, 0.0)$ or $(0.1, 0.5)$ or $(1, 0.0)$ or $(1, 0.5)$
41,42,43,44	$\tau_1 = 21, \tau_2 = 30, A = 5$<br>$(\sigma, \gamma) = (0.1, 0.0)$ or $(0.1, 0.5)$ or $(1, 0.0)$ or $(1, 0.5)$

Table 6: Functional forms and parameter values used to generate simulated non-stationary asymmetric datasets.

Specification of parameters for running existing methods

Let X be the list of time points at which the data has been observed. We have earlier defined `X_range` $= max(X) - min(X)$. Considering that the individual elements of a list are accessed by 1-indexing in R, we additionally define `delta_t` $= X[2] - X[1]$.

MetaCycle's [5] `meta2d` function requires the minimum and maximum period length of interest to be passed as arguments. When running MetaCycle on our simulated datasets, these were respectively specified as

- `minper` $= 2*$`delta_t`
- `maxper` = `X_range`

RAIN's [6] `rain` function requires the period to test for and/or the range of periods to test for to be passed as arguments. These were respectively specified during testing as:

- `period` = `X_range`/2
- `period.delta` = (`X_range`/2)− `delta_t`

For all methods tested that accept the raw data (without merging of replicates) as input, the replicates were passed to the method in an appropriate format.

AUC values of tested methods on simulated datasets

The numbers assigned to simulated datasets in the previous section describing their generation are used here.

Method	AUC value for dataset no.								
	1	2	3	4	5	6	7	8	9
Cosinor	1.000	0.978	0.999	0.960	0.832	0.642	0.796	0.632	0.987
eJTK	1.000	0.954	0.999	0.930	0.766	0.607	0.729	0.586	0.968
GPrank	0.974	0.691	0.943	0.672	0.955	0.649	0.922	0.626	0.679
JTK_Cycle	0.999	0.874	0.994	0.836	0.955	0.720	0.931	0.666	0.897
LSP	0.998	0.811	0.988	0.754	0.942	0.558	0.898	0.545	0.867
Metacycle	0.999	0.873	0.995	0.829	0.962	0.686	0.937	0.642	0.905
ODeGP	0.999	0.911	0.996	0.878	0.996	0.838	0.982	0.786	0.907
SM Kernel	0.983	0.829	0.963	0.782	0.965	0.712	0.922	0.672	0.780
RAIN	0.995	0.847	0.985	0.808	0.985	0.742	0.967	0.709	0.861

Method	AUC value for dataset no.								
	10	11	12	13	14	15	16	17	18
Cosinor	0.608	0.518	0.567	0.516	0.968	0.820	0.884	0.712	0.884
eJTK	0.324	0.444	0.400	0.468	0.995	0.834	0.910	0.720	0.832
GPrank	0.763	0.580	0.649	0.550	0.899	0.678	0.766	0.591	0.962
JTK_Cycle	0.959	0.720	0.800	0.604	0.978	0.777	0.849	0.644	0.818
LSP	0.969	0.680	0.743	0.543	0.967	0.686	0.751	0.540	0.573
Metacycle	0.972	0.725	0.794	0.592	0.982	0.769	0.829	0.621	0.760
ODeGP	0.855	0.698	0.680	0.608	0.946	0.780	0.812	0.657	0.991
SM Kernel	0.723	0.629	0.566	0.559	0.889	0.736	0.653	0.586	0.967
RAIN	0.999	0.828	0.870	0.615	0.994	0.785	0.866	0.628	0.756

Method	AUC value for dataset no.								
	19	20	21	22	23	24	25	26	27
Cosinor	0.716	0.890	0.705	0.927	0.742	0.911	0.727	0.619	0.515
eJTK	0.688	0.834	0.670	0.937	0.723	0.925	0.717	0.405	0.450
GPrank	0.696	0.932	0.695	0.733	0.593	0.721	0.587	0.661	0.562
JTK_Cycle	0.689	0.772	0.675	0.678	0.572	0.660	0.565	0.777	0.634
LSP	0.624	0.542	0.583	0.405	0.572	0.388	0.557	0.558	0.579
Metacycle	0.679	0.710	0.660	0.584	0.580	0.557	0.569	0.719	0.630
ODeGP	0.814	0.966	0.785	0.816	0.665	0.790	0.660	0.787	0.652
SM Kernel	0.753	0.919	0.719	0.506	0.617	0.493	0.599	0.574	0.583
RAIN	0.634	0.746	0.626	0.650	0.532	0.644	0.533	0.801	0.615

Method	AUC value for dataset no.								
	28	29	30	31	32	33	34	35	36
Cosinor	0.620	0.515	0.970	0.845	0.965	0.812	0.684	0.744	0.628
eJTK	0.413	0.461	0.962	0.822	0.937	0.790	0.693	0.723	0.630
GPrank	0.632	0.574	0.783	0.627	0.689	0.842	0.660	0.738	0.594
JTK_Cycle	0.744	0.635	0.898	0.702	0.851	0.903	0.717	0.772	0.614
LSP	0.514	0.540	0.849	0.609	0.778	0.803	0.631	0.659	0.526
Metacycle	0.675	0.617	0.891	0.689	0.848	0.883	0.705	0.745	0.594
ODeGP	0.752	0.634	0.909	0.735	0.871	0.887	0.733	0.766	0.621
SM Kernel	0.566	0.593	0.689	0.644	0.754	0.763	0.679	0.614	0.586
RAIN	0.758	0.610	0.907	0.698	0.820	0.952	0.747	0.806	0.617

Method	AUC value for dataset no.							
	37	38	39	40	41	42	43	44
Cosinor	0.527	0.511	0.516	0.518	0.933	0.776	0.843	0.683
eJTK	0.433	0.496	0.442	0.491	0.950	0.796	0.852	0.688
GPrank	0.788	0.626	0.700	0.597	0.901	0.686	0.768	0.613
JTK_Cycle	0.876	0.678	0.742	0.604	0.958	0.746	0.813	0.641
LSP	0.823	0.636	0.668	0.571	0.944	0.630	0.720	0.526
Metacycle	0.874	0.682	0.727	0.602	0.963	0.730	0.794	0.618
ODeGP	0.787	0.647	0.673	0.586	0.934	0.751	0.808	0.644
SM Kernel	0.646	0.639	0.566	0.575	0.850	0.702	0.627	0.586
RAIN	0.929	0.739	0.778	0.623	0.985	0.776	0.839	0.637

6 Analysis of model complexity penalisation by MLL

The marginal likelihood 17 computed during GP regression automatically incorporates a measure of the complexity of the model being considered through the $\log|K|$ term. A larger $\log|K|$ for a given model generally results from higher covariance values between the data's values at different time points, which produce wider confidence bounds in the GP posterior. Wider bounds allow a greater flexibility of functions in the posterior distribution, indicating that the model is more complex. For this reason, we did not additionally penalise the number of hyperparameters of both models when comparing the two (something that is generally done in regression methods which use the BIC/AIC for model selection). Importantly, we found that the Bayes factor was distinctly different between datasets that were known to be oscillatory versus non-oscillatory, thus highlighting the usefulness of this metric in the classification problem.

The expression for K_{NS} in 15 contains a variable Q, which denotes the number of components [7] of K_{NS}. Every increase of 1 in Q adds 9 new hyperparameters to the defined K_{NS}. We show below that increasing Q (and thus the number of hyperparameters) does not cause overfitting of the data in the posterior generated by K_{NS}, and that the MLL does penalise the more 'complex' resulting model represented by K_{NS}.

Non-oscillatory data was simulated by sampling from $\mathcal{N}(0,1)$ at 3 hour intervals for a duration of 48 hours, and generating three replicates from this base waveform as described above. ODeGP was run on this data with multiple values of Q. The datafit and complexity term of the MLL for K_{NS} were respectively calculated as $-0.5Y^T K_{XX}^{-1} Y$ and $-0.5\log K_{XX}$, as in 17.

Number of components (Q) used in K_{NS}	Total number of hyperparameters	Bayes factor	Datafit term (K_{NS})	Complexity term (K_{NS})
1	10	2.2397	-7.9760	1.6739
2	19	1.1253	-5.7758	-1.2145
3	28	0.5657	-7.5851	-0.0930
4	37	0.1715	-6.6950	-2.1765
5	46	0.1056	-8.1752	-1.1811
6	55	0.0438	-8.3841	-1.8528

Table 7: Comparison of the Bayes factor and individual terms of the MLL for K_{NS}, obtained by testing ODeGP on the non-oscillatory data shown in 2 with different values of Q in K_{NS}. A higher value of the datafit term indicates a closer fit of the data by the K_{NS} posterior mean, and a higher value of the complexity term indicates a less complex (and more favourable) model generated by K_{NS}.

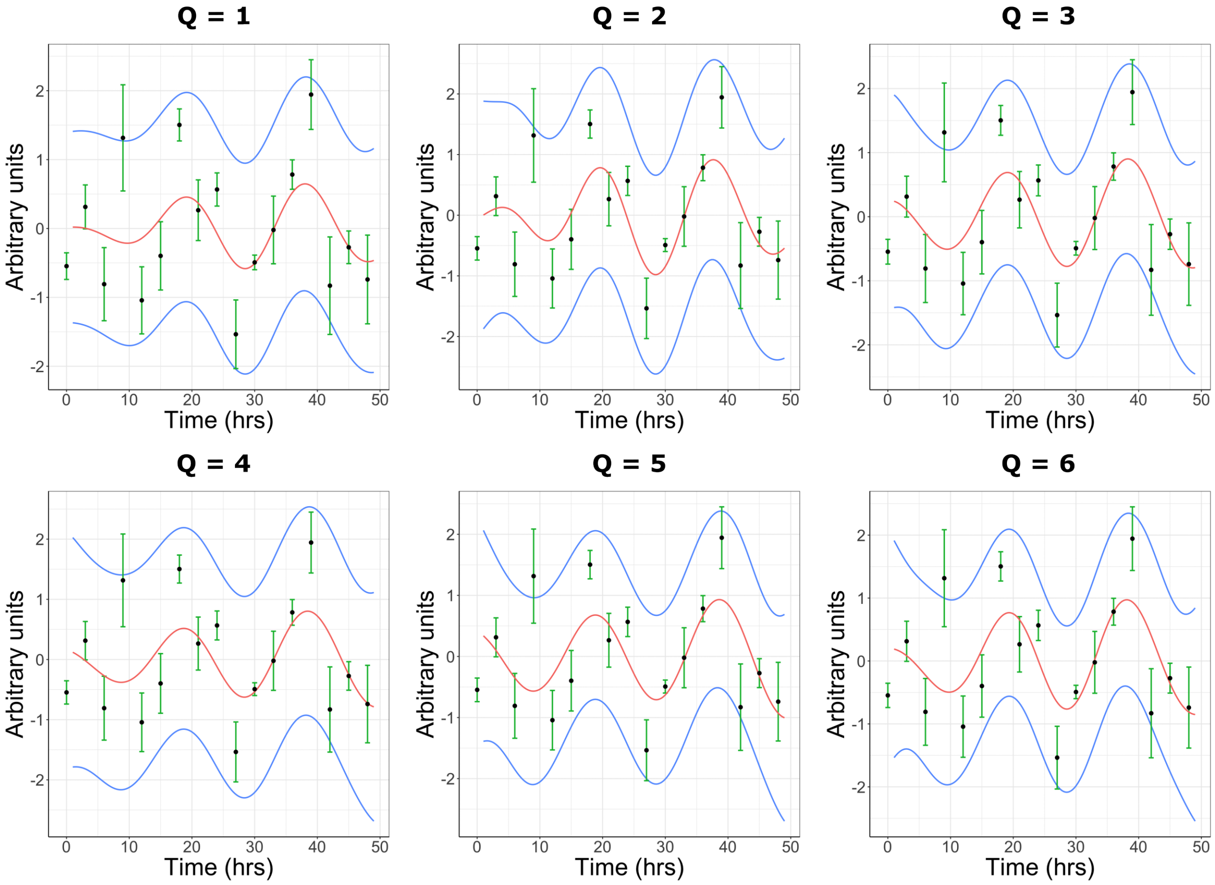

Figure 2: Posterior distributions of the non-stationary kernel with varying number of components Q, obtained by application of ODeGP on simulated non-oscillatory data.

Figure 2 demonstrates that increasing the number of hyperparameters used in the non-stationary kernel does not enforce a closer fitting of the data by the posterior mean. This is also reflected in the datafit term of K_{NS} in Table 7. The complexity term worsens as well (though not monotonically) and there is a clear trend of reduction in the Bayes factor. Overall, a larger Q leads to a stronger indication of K_D representing a better model for the data.

7 Application of ODeGP to qPCR datasets

To understand how well ODeGP works on existing qPCR datasets, we analyzed gene expression profiles reported in a prior work [8]. While the expression patterns and analysis of *Rev-ERBβ* and *Per1* were shown in the main text Figure 4, here we present analysis of some of the other reported genes, *Per2* and *Npas2* along with the unsynchronized data for *Per1*. It is clear that between the Dex and vehicle treated datasets, the Bayes factor is always separated by an order of magnitude or more, demonstrating the power of ODeGP in classifying oscillatory datasets.

Similar to the analysis of the synchronized *Per1* data shown in Table 4 of the main text, we also evaluated the Bayes factor bounds produced by each method for the unsynchronized Vehicle-treated *Per1* data in Figure 3C. The results of this are shown in Table 8. Barring MetaCycle and RAIN, all methods correctly classify the data as non-oscillatory with p-values greater than 0.05. The Bayes factor bounds obtained for the remaining methods are all sufficiently close to 1, strongly indicating that the data does not contain oscillations. ODeGP's non-stationary kernel posterior for this data in Figure 3C shows very wide confidence intervals, confirming that this

kernel is not a good model for it.

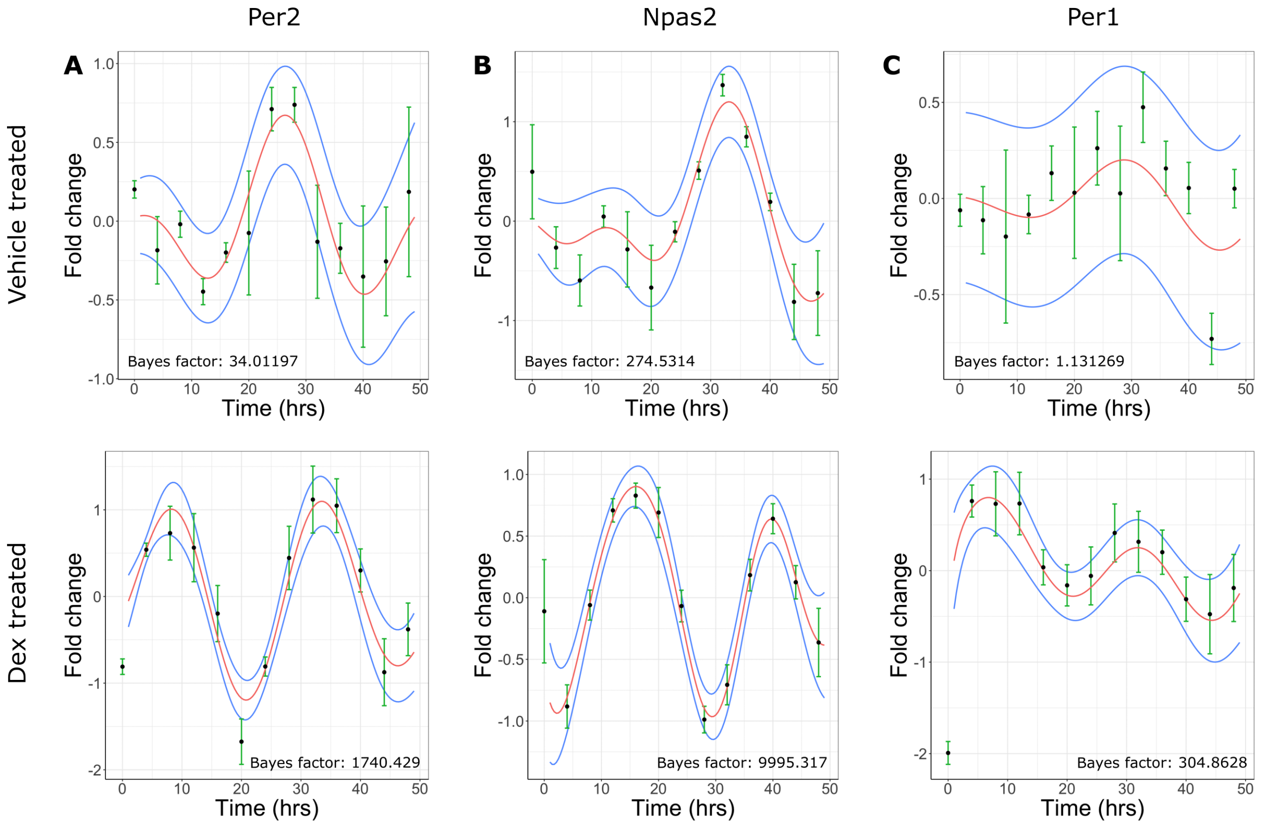

Figure 3: Application of ODeGP on *Per2*, *NPas2* and *Per1* expression data measured using qPCR [8]. (A) The posterior distributions (mean in red, standard deviation in blue) obtained by defining a GP prior using the non-stationary kernel, and performing GP regression on the relative gene expression of *Per2* after treatment with DMSO (above) or Dexamethasone (below), measured at 4 hour intervals over a 48 hour duration and averaged over 3 biological replicates. (B) The GP posteriors of the non-stationary kernel applied to relative gene expression of *Npas2* when treated with DMSO (above) or Dexamethasone (below), where the posterior mean and standard deviation are in red, blue respectively. (C) The GP posteriors of the non-stationary kernel applied to relative gene expression of *Per1* when treated with DMSO (above) or Dexamethasone (below), where the posterior mean and standard deviation are in red, blue respectively.

Method	Metric type	Metric value	Bayes factor (bound)
Cosinor	Q-value	0.50131	1.0000
eJTK	p-value	0.99289	1.0000
GPrank	log of Bayes factor	0.09877	1.1038
JTK_Cycle	p-value	0.09852	1.6112
LSP	p-value	0.11609	1.4716
MetaCycle	p-value	0.01397	6.1659
ODeGP	Bayes factor	1.1313	1.1313
SM Kernel	Bayes factor	1.3785	1.3785
RAIN	p-value	0.00617	11.7184

Table 8: Comparison of output metrics of all methods tested on the Vehicle-treated *Per1* data shown in Figure 3C. p-values/Q-values were converted to corresponding Bayes factor bound [9] values where applicable to allow comparison with the Bayes factor returned by ODeGP.

8 Comparison of ODeGP with other GP based algorithms

Though not explored extensively, there are a few prior examples of the use of GPs in modeling biological data, for example in identifying differentially expressed genes [3, 10], detecting oscillations [11, 12] and the discovery of spatial patterns in gene expression [13]. The method in [12] uses a principle similar to ours with the comparison of a non-oscillatory GP model to an oscillatory GP model. However, this method does not explicitly incorporate a non-stationary functional form to encode correlations between the data at different time points in its oscillatory model, and also compares its performance only with that of the LS Periodogram (which never falls among the best 3 performing methods in our analysis). GPrank [10] on the other hand uses GPs to model genome-wide time series data. Though it was not created for the specific purpose of detecting oscillations, the workflow followed by this method is similar to ours in terms of the combination of two GP models with Bayesian model selection. The significant difference between GPrank and ODeGP is in the choice of kernel used to define the alternate hypothesis (the RBF kernel is used in GPrank). Our results show that ODeGP almost always outperforms GPrank (Tables 2 and 3), demonstrating the importance of careful choice of the kernel for the specific problem at hand. This point also highlights the flexibility of GPs in modeling various kinds of datasets simply by appropriate choice of kernel functions. Similar to GPrank, the output metric used by ODeGP to make a decision on the presence of oscillations is the Bayes factor, which is a ratio of the marginal likelihoods of the competing models.

9 Modeling coupled oscillators to investigate the role of amplitude and coupling strength on the Bayes Factor

We used a Poincare model of coupled phase-amplitude oscillators [14] to investigate how the Bayes Factor from ODeGP is affected by increasing the amplitude of single cell oscillators as well as coupling between them. Converting radial coordinates of the ith oscillator r_i, θ_i to Cartesian coordinates x_i, y_i with $r_i = \sqrt{x_i^2 + y_i^2}$, we get the following equations for the Poincare oscillator:

$$\frac{dx_i}{dt} = F(\bar{x}, \bar{y}) = \gamma_i x_i (A_i - r_i) - \frac{2\pi}{\tau_i} y_i + M, \tag{23}$$

$$\frac{dy_i}{dt} = G(\bar{x}, \bar{y}) = \gamma_i y_i (A_i - r_i), \tag{24}$$

$$\text{where } M = \frac{K}{N} \sum_{i=1}^{N} x_i(t). \tag{25}$$

Here N is the total number of single cell oscillators which are coupled via the mean field M, with the coupling strength parameterised by K. The free-running time period of the ith oscillator is τ_i. All oscillators were assigned the same amplitude $A_i = A$, and the amplitude relaxation rate of the ith oscillator is γ_i. $x_i(t)$ and $y_i(t)$ were computed using the Euler–Maruyama method, solving the Equations 23 and 24 over time interval $[0, T]$ with timestep Δt.

$$x_i(t + \Delta t) = x_i(t) + \Delta t \cdot F(\bar{x}, \bar{y}) + \sigma\sqrt{\Delta t} \cdot \mathcal{N}(0, 1) \quad (26)$$

$$y_i(t + \Delta t) = y_i(t) + \Delta t \cdot G(\bar{x}, \bar{y}) + \sigma\sqrt{\Delta t} \cdot \mathcal{N}(0, 1) \quad (27)$$

All oscillators were given the same initial condition $x_i(0) = x(0)$ with $y_i(0) = y(0) = \sqrt{A^2 - x(0)^2}$, where $x(0)$ was drawn from a uniform distribution $U(0, A)$. Other parameters were set as follows: $N = 1000$, $T = 1440$, $\Delta t = 1$, and $\gamma_i = \gamma = 0.1$. The time period of the ith oscillator, τ_i, was drawn from a normal distribution $\mathcal{N}(24, 2)$.The mean $\mu_x(t)$ and standard deviation $\sigma_x(t)$ of the computed $x_i(t)$ over $i \in \{1, 2, \dots, N\}$, were found for $t \in \{0, 1, \dots, T\}$. $\mu_x(t)$ and $\sigma_x(t)$ were then sampled at 3-hour intervals over a 48-hour duration from $t = 1200$ onwards, to provide sufficient time after $t = 0$ for the system of oscillators to reach steady state. These sampled time-series were input to ODeGP with $\mu_x(t)$ as the data and $\sigma_x(t)$ as the error-bar values, to obtain the corresponding Bayes factor. This process was repeated for all combinations of $A \in \{1, 1.5, 2, \dots, 5\}$, $K \in \{0, 0.005, 0.01, \dots, 0.2\}$, and $\sigma \in \{0, 0.05, 0.1, \dots, 0.5\}$. The variation of the produced Bayes factor with A and K for each value of σ is shown in Figure 4.

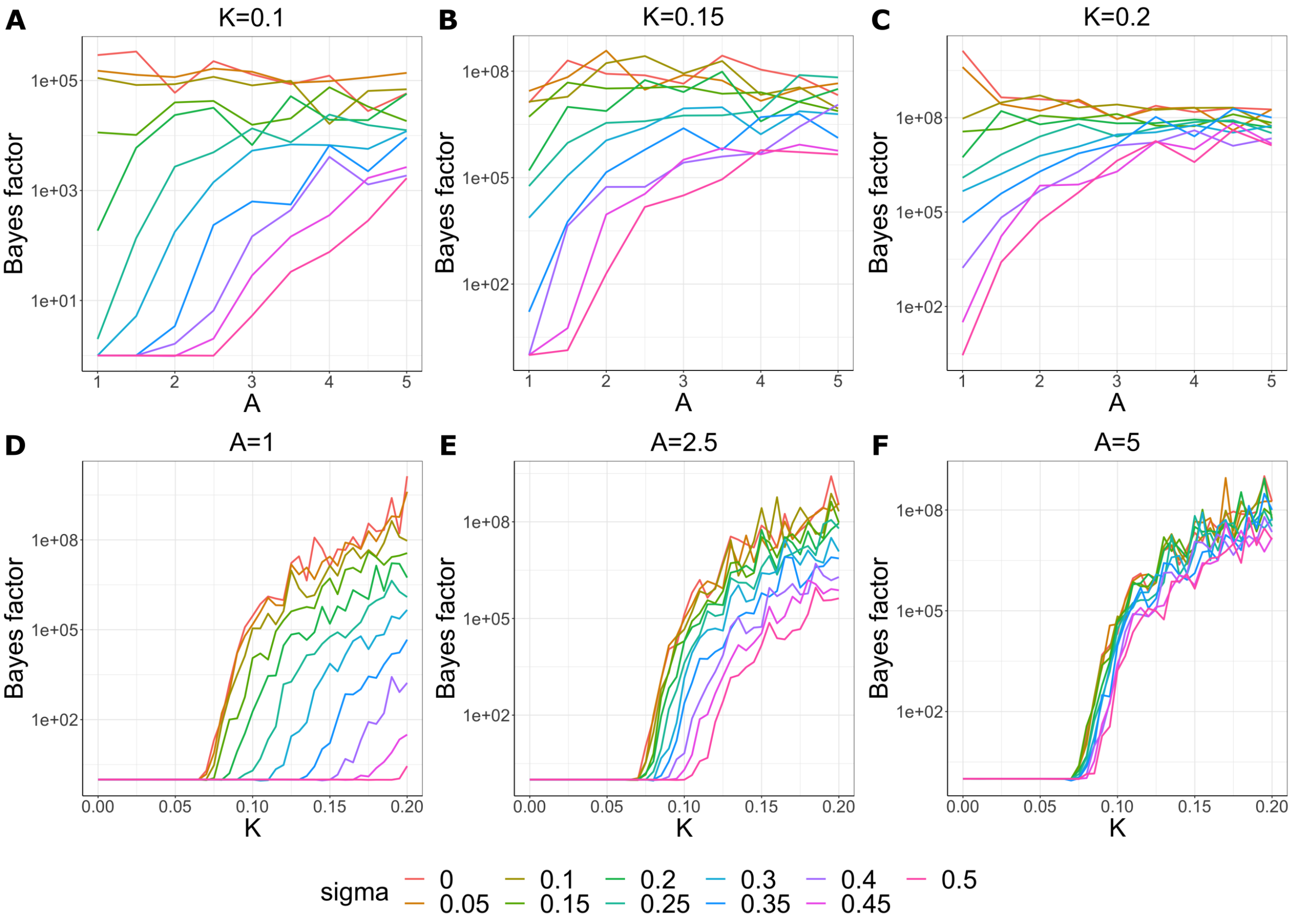

Figure 4: Variation of the Bayes factor obtained by applying ODeGP on simulations of the Poincare model of coupled oscillators. (A)–(C) Bayes factor as a function of the amplitude A for fixed values of coupling strength $K = 0.1, 0.15$ and 0.2. (D)–(F) Bayes factor as a function of K for fixed values of $A = 1, 2.5$ and 5. In all panels, the different colours represent different noise strengths σ.

References

[1] Carl Edward Rasmussen and Christopher K. I. Williams. *Gaussian Processes for Machine Learning*. Google-Books-ID: Tr34DwAAQBAJ. MIT Press, Nov. 2005.

[2] Katharine Mullen et al. "DEoptim: An R Package for Global Optimization by Differential Evolution". In: *Journal of Statistical Software* 40.6 (2011), pp. 1–26.

[3] Markus Heinonen et al. "Detecting time periods of differential gene expression using Gaussian processes: an application to endothelial cells exposed to radiotherapy dose fraction". In: *Bioinformatics* 31.5 (Mar. 2015), pp. 728–735.

[4] Peter H. Westfall, Wesley O. Johnson, and Jessica M. Utts. "A Bayesian Perspective on the Bonferroni Adjustment". In: *Biometrika* 84.2 (1997). Publisher: [Oxford University Press, Biometrika Trust], pp. 419–427.

[5] Gang Wu et al. "MetaCycle: an integrated R package to evaluate periodicity in large scale data". In: *Bioinformatics* 32.21 (July 2016), pp. 3351–3353. eprint: `https://academic.oup.com/bioinformatics/article-pdf/32/21/3351/7889685/btw405.pdf`.

[6] Paul F. Thaben and Pål O. Westermark. "Detecting Rhythms in Time Series with RAIN". In: *Journal of Biological Rhythms* 29.6 (Oct. 2014), pp. 391–400.

[7] Sami Remes, Markus Heinonen, and Samuel Kaski. "Non-Stationary Spectral Kernels". In: (2017).

[8] Alex Y.-L. So et al. "Glucocorticoid regulation of the circadian clock modulates glucose homeostasis". In: *Proceedings of the National Academy of Sciences of the United States of America* 106.41 (Oct. 2009), pp. 17582–17587.

[9] Daniel J. Benjamin and James O. Berger. "Three Recommendations for Improving the Use of p-Values". In: *The American Statistician* 73 (2019), pp. 186–191. eprint: `https://doi.org/10.1080/00031305.2018.1543135`.

[10] Hande Topa and Antti Honkela. "GPrank: an R package for detecting dynamic elements from genome-wide time series". In: *BMC Bioinformatics* 19.367 (2018).

[11] Nicolas Durrande et al. "Detecting periodicities with Gaussian processes". In: *PeerJ Comput. Sci.* 2.e50 (Apr. 2016), e50.

[12] Nick E Phillips et al. "Identifying stochastic oscillations in single-cell live imaging time series using Gaussian processes". In: *PLoS Comput. Biol.* 13.5 (May 2017), e1005479.

[13] Valentine Svensson, Sarah A. Teichmann, and Oliver Stegle. "SpatialDE: identification of spatially variable genes". In: *Nature Methods* 15.5 (May 2018). Number: 5 Publisher: Nature Publishing Group, pp. 343–346.

[14] Christoph Schmal, Erik D Herzog, and Hanspeter Herzel. "Measuring Relative Coupling Strength in Circadian Systems". In: *Journal of Biological Rhythms* 33.1 (2018). PMID: 29219034, pp. 84–98. eprint: `https://doi.org/10.1177/0748730417740467`.
